# Phenolic Derivatives of *Astragalus aitosensis* with Selective MAO-B Inhibition and Mitochondrial Protection

**DOI:** 10.3390/molecules30204069

**Published:** 2025-10-13

**Authors:** Preslav Enchev, Magdalena Kondeva-Burdina, Emilio Mateev, Iliana Ionkova, Yancho Zarev

**Affiliations:** 1 Department of Pharmacognosy, Faculty of Pharmacy, Medical University of Sofia, 2 Dunav Str., 1000 Sofia, Bulgaria; p.enchev@pharmfac.mu-sofia.bg (P.E.); ionkova@pharmfac.mu-sofia.bg (I.I.); 2 Department of Pharmacology, Pharmacotherapy and Toxicology, Faculty of Pharmacy, Medical University of Sofia, 2 Dunav Str., 1000 Sofia, Bulgaria; mkondeva@pharmfac.mu-sofia.bg; 3 Department of Pharmaceutical Chemistry, Faculty of Pharmacy, Medical University of Sofia, 2 Dunav Str., 1000 Sofia, Bulgaria; e.mateev@pharmfac.mu-sofia.bg

**Keywords:** *Astragalus aitosensis*, phenolic derivatives, MAO-B

## Abstract

*Astragalus aitosensis*, also known as *Astracantha arnacantha* (M. Bieb.) Podlech subsp. *aitosensis* (Ivanisch.) Réer & Podlech, a Bulgarian endemic species, was investigated for its phenolic profile and neuroprotective potential. A targeted extraction approach led to the isolation of 14 phytochemicals. According to our literature review, none of the isolated chemicals have been reported before for *A. aitosensis*. Two of them are previously undescribed molecules—an isomer of odoratin and 6-hydroxy-3-(2-hydroxy-4-methoxyphenyl)-7-methoxy-4*H*-1-benzopyran-4-one—and four of them had not been observed before our study in the genus *Astragalus*: 3′-methoxydaidzein, fujikinetin, sayanedine, and 6,4′-dimethoxy-7,2′-dihydroxyisoflavone. Five of the phytochemicals—maackiain, cajanin, onogenin, afrormosin, and sayanedine—exhibited selective inhibition of human monoamine oxidase-B (MAO-B), with maackiain reducing activity by 45%, nearing the effect of selegiline. The investigated phytochemicals also showed significant antioxidant and neuroprotective effects in ex vivo models using isolated rat brain synaptosomes, mitochondria, and microsomes, mitigating oxidative stress by preserving glutathione levels and reducing lipid peroxidation. Molecular docking confirmed favorable binding of active phytochemicals, particularly maackiain, within the MAO-B active site. Structure–activity relationship (SAR) analysis highlighted the role of specific substituents and fused-ring systems in MAO-B inhibition. This study expands our knowledge of the phytochemical diversity of *A. aitosensis* and supports the therapeutic relevance of its phenolic compounds in neurodegenerative disorders such as Parkinson’s disease.

## 1. Introduction

The genus *Astragalus* L. (Fabaceae), comprising nearly 2900 species, is the world’s largest vascular plant group, widely valued for its medicinal, nutritional, and practical applications [1,2]. *Astragalus aitosensis*, also known as *Astracantha arnacantha* (M. Bieb.) Podlech subsp. *aitosensis* (Ivanisch.) Réer & Podlech, is a local endemic subspecies found in the territory of Bulgaria. It is classified as a tertiary relic, with a conservational status of critically endangered [3,4,5,6]. The plant is a small spiny shrub that thrives in transitional/continental climates on rocky, eroded slopes with poor soils, forming part of a unique ecosystem of conservation significance. It typically grows to a height of 30–50 cm, with strongly branched stems. Flowering occurs from May to June, with fruiting following in July and August. Reproduction is achieved both by seeds and vegetative means, allowing the species to adapt to its challenging environment. These habitats, endangered by urbanization and land use changes, require protection to preserve their biodiversity and ecological value [6].

Major classes of biologically active compounds identified in this genus include saponins and polyphenols, some of which are rare metabolites [7,8,9,10]. Polyphenols, a significant class of bioactive compounds in the genus *Astragalus*, are synthesized via the phenylpropanoid pathway, a central metabolic route in plants. The pathway generates diverse secondary metabolites, including lignins, pterocarpanes, flavonoids, and isoflavonoids, which play essential roles in plant growth, development, and environmental adaptability. Flavonoids, characterized by their diphenylpropane (C_6_-C_3_-C_6_) skeleton, represent a key branch of this pathway typical for the aerial parts of the plant, encompassing subclasses, such as chalcones, flavones, pterocarpanes, flavonols, and isoflavones [11].

From a pharmacological perspective, many studies have demonstrated the actions of isoflavonoids through diverse mechanisms, exhibiting effects such as anti-cancer, anti-inflammatory, and phytoestrogenic activities, with compounds like genistein, calycosin, and ononin playing prominent roles. Genistein demonstrates strong anti-cancer potential by inhibiting the PI3K/AKT pathway, promoting tumor cell apoptosis, and suppressing EGF-mediated proliferation, particularly in lung cancer [12,13]. Ononin, a glucoside of formononetin found in *A. mongholicus*, exhibits antiproliferative and pro-apoptotic effects in various cancers, including breast and non-small cell lung cancer (NSCLC), by downregulating the PI3K/AKT/mTOR signaling pathway and enhancing apoptosis markers like caspase-3 and caspase-9. Additionally, calycosin has been highlighted for its anti-cancer and anti-inflammatory properties, showing significant activity, and has been described as the most effective *A. membranaceus* compound for conditions like gastric cancer and kidney diseases [14,15,16]. Within the *Astragalus* genus, phenolic metabolites with reported MAO activity include maackiain and formononetin. Maackiain is a potent, selective human MAO-B inhibitor (sub-µM IC_50_) and exemplifies the pterocarpan chemotype’s fit to the MAO-B aromatic cage, while formononetin shows modest inhibition with MAO-B IC_50_ ≈ 11.0 µM and MAO-A IC_50_ ≈ 21.2 µM in enzyme assays [17,18] In line with this scaffold trend, medicarpin and homopterocarpin (closely related pterocarpans) also display strong MAO-B inhibition (IC_50_ ≈ 0.45–0.72 µM, human MAO-B), providing SAR context for the high activity observed for maackiain [19]. By contrast, for cajanin, we did not locate prior experimental MAO-A/B enzyme data in primary reports or recent reviews (only broader discussions of flavonoid MAOIs), whereas onogenin and pseudobaptigenin have in silico flags (ChemGPS-NP/QSAR) as candidate MAO-B inhibitors pending wet-lab confirmation [20,21].

The genus *Astragalus* remains relatively unexplored regarding its isoflavonoid content. *A. membranaceus*, a key plant in the Chinese Materia Medica and pharmacopeia, is widely used in various treatments and has yielded an enormous number of isolated phytochemicals from different classes [2]. The most common isoflavonoids isolated from different *Astragalus* species are formononetin, calycosin, cajanin, and ononin from species like *A. taipaishanensis, A. cicer, A. peregrinus,* and *A. complanatus* [8,22,23,24,25,26,27,28]. Our study focuses on the isolation and identification of biologically active metabolites from the aerial parts of *A. aitosensis*, which has been previously investigated only to a limited extent, primarily in terms of its flavonoid composition. Identified structures include isorhamnetin-3-*O*-robinobioside, alangiflavoside, quercetin, and kaempferol [29,30].

## 2. Results

### 2.1. Identification of Isolated Substances

**Figure 1:**
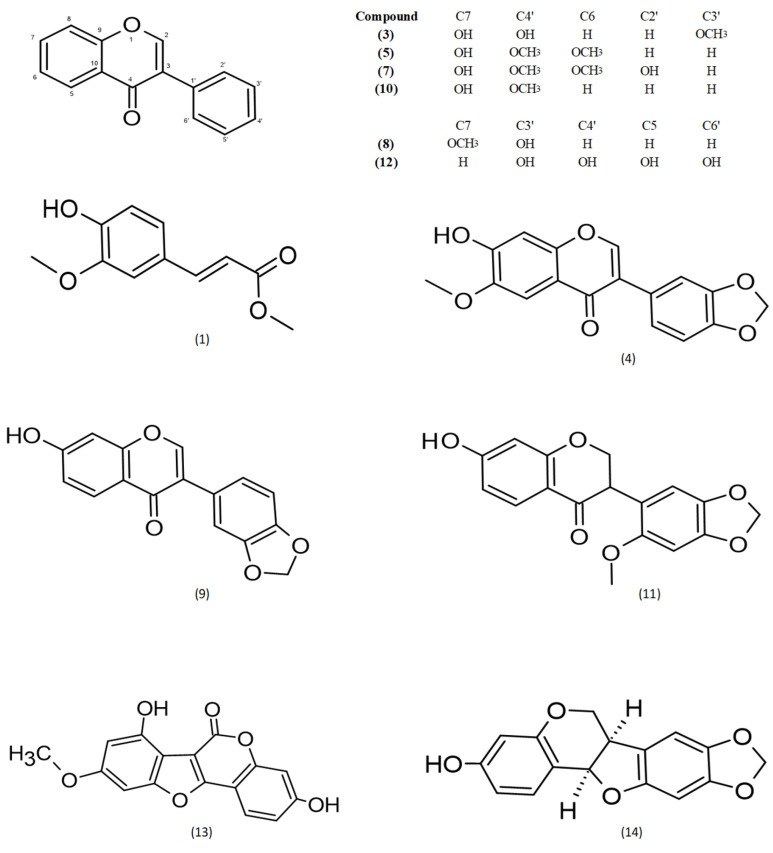
Chemical structures of compounds **1**–**14**.

By means of semi-prep HPLC purification of fractions A3, A4 and A5, a series of known isoflavonoids were isolated. The structures of the known compounds were identified based on previously described data: (**1**) methylferulate [31], (**3**) 3′-methoxydaidzein [32], (**4**) fujikinetin [33,34], (**5**) afrormosin [34,35], (**7**) 6, 4′-dimethoxy-7, 2′-dihidroxy [36], (**8**) sayanedine [37], (**9**) pseudobaptigenin [38], (**10**) formononetin [39], (**11**) onogenin [40,41], (**12**) cajanin [42,43] (**13**) trifoliol [44] and (**14**) maackiain [45,46] (Figure 1). Chromatograms and MS spectra are presented in Supplementary Materials Figures S1–S13.

**Figure 2:**
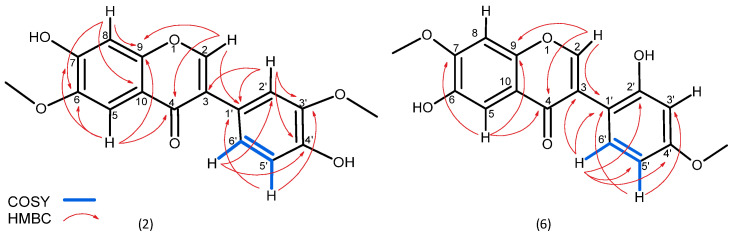
COSY and HMBC correlations for structures **2** and **6**.

In addition, two more metabolites were isolated. Compound **2** was isolated as a yellowish powder, which displayed a protonated molecular ion at *m*/*z* 315.0858 [M + H]^+^, corresponding to a molecular formula C_17_H_15_O_6_^+^ (calcd. 315.0863) (Supplementary Materials Figure S13). As for most of the identified substances, the ^1^H NMR spectrum of substance **2** revealed a characteristic singlet for isoflavones at δ_H_ 8.36. COSY correlations between H-5′ (δ_H_ 6.80) and H-6′ (δ_H_ 6.99) and coupling constants between H-6′ (*J* 8.18, 2.05 Hz) and H-2′ (*J* 2.0 Hz) (Table 1) confirmed a meta and para substation in the B ring (Figure S15). In addition to this substation are HMBC correlations observed between C-3′ (δ_C_ 147.4) and the protons at H-2′ and H-5′, as well as between C-4′ (δ_C_ 146.5) and the protons at H-2′ and H-6′ and especially the correlation between C-1′ (δ_C_ 123.1) and the proton at H-2′. As expected, the position of methoxylations was confirmed by the long-range correlation of C-3′ (δ_C_ 147.4) with the proton of the methoxy group (δ_H_ 3.78). The two singlets at H-5 (δ_H_ 7.38) and H-8 (δ_H_ 7.15) confirm a substitution for the A ring at positions C-6 and C-7. The position of the second methoxy group is revealed by the long-range HMBC correlation observed between C-6 (δ_C_ 153.8) and the singlet protons at δ_H_ 3.91 (NMR spectra for **2**—Figures S14–S17 of the Supplementary Materials). Therefore, the structure of **2** was defined as a positional isomer of odoratin (Figure 2). 

Substance **6** was isolated as a white powder observed as a protonated molecular ion with *m*/*z* 315.0858, corresponding to the same molecular formula C_17_H_15_O_6_^+^ (calcd. 315.0863) (Figure S18) as compound **2**. Similarly to **2**, the NMR spectra of 6 revealed the presence of a characteristic singlet for isoflavones at δ_H_ 8.23 and the same pattern of substitution in ring A (two singlets at H-5 (δ_H_ 7.39) and H-8 (δ_H_ 6.86)). The different position of methoxylation is confirmed by the long-range HMBC correlation of C-7 (δ_C_ 147.5) with the proton of the methoxy group (δ_H_ 3.86) (Figure S23). The same pattern of proton arrangement in ring B as for substance **2** is also observed for 6 (H-5′ (δ_H_ 7.03), H-6′ (δ_H_ 6.94) and H-3′ (δ_H_ 6.94)) but with a difference in the HMBC correlations. The lack of expected correlations for C-1′ (δ_C_ 122.9) relocates the substitutions in ring B as orto and para. Similarly, the position of methoxylations in ring B was confirmed again by the long-range correlation of C-4′ (δ_C_ 147.5) with the proton of the methoxy group (δ_H_ 3.78) NMR spectra are presented in Figures S18–S23 in the Supplementary Materials. Thus, compound 6 is described here for the first time as 6-hydroxy-3-(2-hydroxy-4-methoxyphenyl)-7-methoxy-4*H*-1-benzopyran-4-one (Figure 2).

### 2.2. Effects on Human Recombinant MAO-A/MAO-B Enzyme (hMAO-A/hMAO-B)

**Figure 3:**
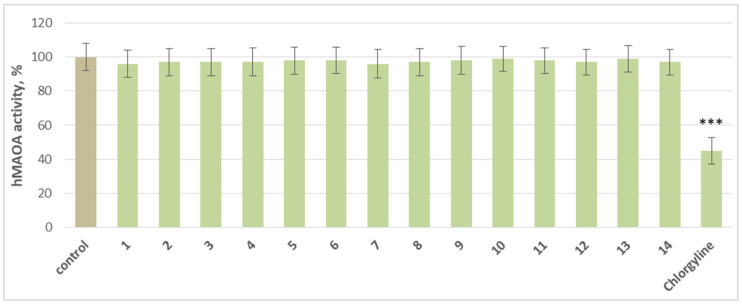
Effects of the tested substances and chlorgyline, applied individually at a concentration of 1 µM, on hMAO-A enzyme activity. *** *p* < 0.001 vs. control (pure hMAO-A).

At a concentration of 1 µM, the individual compounds did not produce a statistically significant inhibition of hMAO-A activity compared to the control. Only the classical MAO-A inhibitor chlorgyline significantly reduces enzyme activity by 55% compared to the control (pure hMAO-A) (Figure 3).

**Figure 4:**
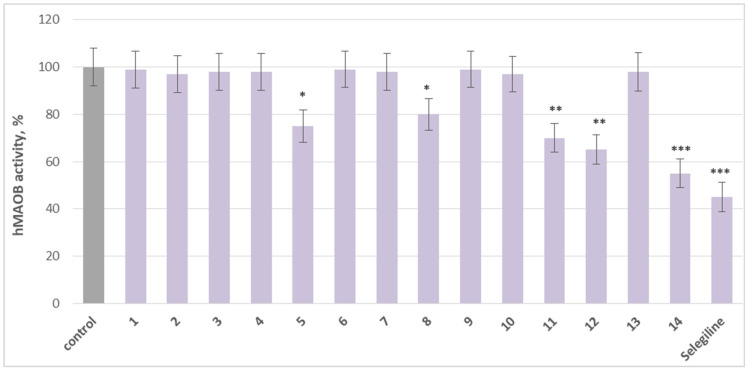
Comparative effects of the tested agents and selegiline, each applied individually at a concentration of 1 µM on hMAO-B activity. * *p* < 0.05; ** *p* < 0.01; *** *p* < 0.001 vs. control (pure hMAO-B).

On the other hand, **5** (afrormosin), (sayanedine), **11** (onogenin), **12** (cajanin), and **14** (maackiain) exhibit significant MAO-B inhibitory activity compared to the control (pure hMAO-B). Substance **5** reduced MAO-B activity by 25%, compound **8** by 20%, compound **11** by 30%, and **12** by 35%, relative to the control. Maackiain (**14**) exhibited the strongest inhibition, decreasing MAO-B activity by 45%, approaching the effect of selegiline, a classical MAO-B inhibitor, which reduced activity by 55% (Figure 4).

The substances with statistically significant MAO-B inhibitory activity (**5**, **8**, **11**, **12** and **14**) were further evaluated for potential neurotoxicity when applied individually and for neuroprotection in different neurotoxicity models using isolated rat brain subcellular fractions (synaptosomes, brain mitochondria, and microsomes).

### 2.3. Effects on Isolated Rat Brain Synaptosomes

**Figure 5:**
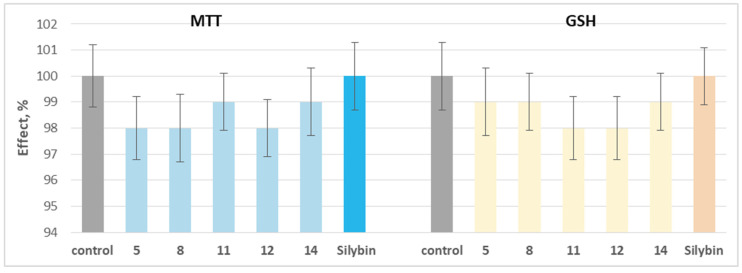
Effects of compounds **5**, **8**, **11**, **12**, and **14**, as well as silybin, applied individually at a concentration of 100 µM, on synaptosomal viability and GSH levels in isolated rat brain synaptosomes.

When applied individually at a concentration of 100 µM, the tested phytochemicals and silybin did not exhibit statistically significant neurotoxic effects on isolated rat brain synaptosomes. They did not significantly alter the key parameters characterizing the functional–metabolic status of synaptosome–synaptosomal viability (determined by the MTT test) and reduced glutathione (GSH) levels (Figure 5).

### 2.4. Neuroprotective and Antioxidant Effects

**Figure 6:**
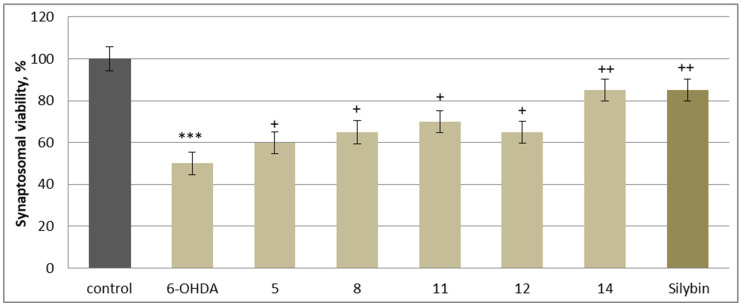
Effects of **5**, **8**, **11**, **12**, **14** and silybin at a concentration of 50 µM in combination with 6-OHDA on synaptosomal viability. *** *p* < 0.001 vs. control (untreated synaptosomes); ^+^ *p* < 0.05; ^++^ *p* < 0.01 vs. control (pure 6-OHDA).

**Figure 7:**
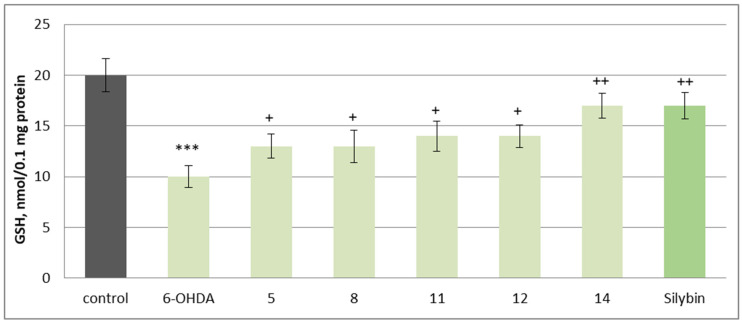
Effects of **5**, **8**, **11**, **12**, **14**, and silybin at a concentration of 50 µM in combination with 6-OHDA on GSH levels in isolated rat brain synaptosomes. *** *p* < 0.001 vs. control (untreated synaptosomes); ^+^ *p* < 0.05; ^++^ *p* < 0.01 vs. control (pure 6-OHDA).

In this neurotoxicity model, substances **5**, **8**, **11**, **12**, and **14**, at a concentration of 50 µM, exhibited statistically significant neuroprotective and antioxidant activity compared to the control (toxic agent). When applied alone at a concentration of 150 µM, 6-OHDA significantly reduced synaptosomal viability and GSH levels by 50% compared to the control (untreated synaptosomes) (Figure 6 and Figure 7). When co-administered with 6-OHDA, substances **5**, **8**, **11**, **12**, **14** and silybin, at a concentration of 50 µM, preserved synaptosome viability and GSH levels as follows: **5** preserved synaptosome viability by 20%; **8** and **12** preserved it by 30%; **11** by 40%; **14** and silybin preserved this parameter by 60% compared to the control (pure 6-OHDA) (Figure 6).

Substances **5** and **8** preserve the level of reduced glutathione by 30%, **11** and **12** by 40%, while **14** and silybin preserve it by 70% compared to the control (pure 6-OHDA) (Figure 7).

The possible mechanism of neuroprotective action in this neurotoxicity model is most likely related, on one hand, to the inhibition of MAO-B activity and, on the other, to the preservation of reduced glutathione levels, the primary nucleophile responsible for scavenging free radicals. It has been established that oxidative stress is one of the main mechanisms through which neurotoxic substances induce apoptosis or necrosis [47].

### 2.5. Effects on Isolated Rat Brain Mitochondria

**Figure 8:**
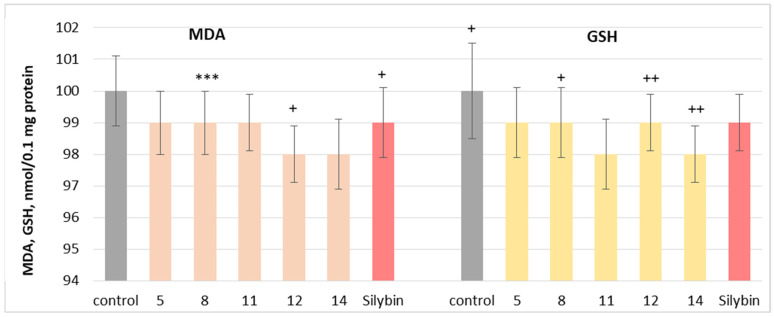
Effects of **5**, **8**, **11**, **12**, **14** and silybin, applied individually at a concentration of 100 µM, on GSH levels in isolated rat brain mitochondria. *** *p* < 0.001 vs. control (untreated synaptosomes); ^+^ *p* < 0.05; ^++^ *p* < 0.01 vs. control.

The neuroprotective potential of the isolated phytochemicals was further evaluated using a mitochondrial oxidative stress model induced by t-BuOOH. Oxidative stress is a key mechanism through which neurotoxic compounds induce apoptosis and necrosis, making it a widely used model for assessing mitochondrial dysfunction [47]. When applied individually at 100 µM, substances **5**, **8**, **11**, **12** and **14** and silybin did not exhibit statistically significant neurotoxic effects on rat brain mitochondria. They did not significantly alter the key metabolic biomarkers of mitochondrial function, such as malondialdehyde (MDA) production and reduced glutathione (GSH) levels, suggesting good mitochondrial tolerance (Figure 8).

**Figure 9:**
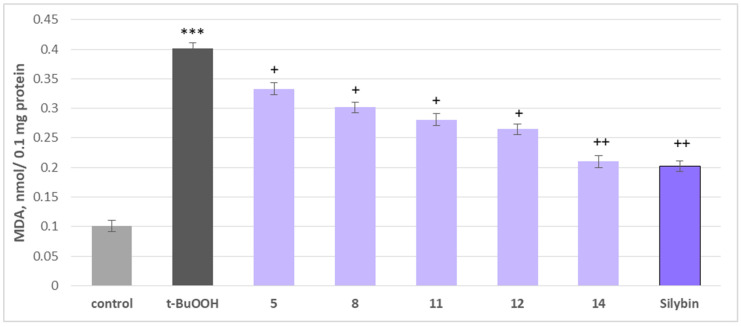
Effects of **5**, **8**, **11**, **12, 14**, and silybin at a concentration of 50 µM, in combination with t-BuOOH, on MDA production in isolated rat brain mitochondria. *** *p* < 0.001 vs. control (untreated mitochondria), ^+^ *p* < 0.05; ^++^ *p* < 0.01 vs. control (pure t-BuOOH).

To assess their potential protective effects, the substances were tested against t-BuOOH-induced oxidative stress. When applied alone at 75 µM, t-BuOOH significantly increased MDA levels by 297% and reduced GSH levels by 50%, confirming its role as a strong oxidative stress inducer. However, when co-administered at 50 µM, phytochemicals **5**, **8**, **11**, **12**, **14** and silybin effectively mitigated mitochondrial damage (Figure 9).

**Figure 10:**
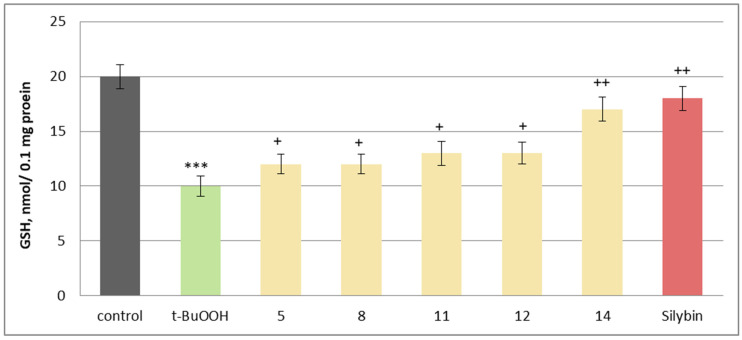
Effects of **5**, **8**, **11**, **12**, **14**, and silybin at a concentration of 50 µM, in combination with t-BuOOH, on GSH levels in isolated rat brain mitochondria. *** *p* < 0.001 vs. control (untreated mitochondria), ^+^ *p* < 0.05; ^++^ *p* < 0.01 vs. control (pure t-BuOOH).

The most pronounced effects were observed with maackiain (**14**) and silybin, which reduced MDA production by 48% and 50%, respectively, while preserving GSH levels by 70% and 80%, compared to the toxic agent alone. Other active investigated substances, including onogenin (**11**) and cajanin (**12**), also showed notable protective effects, reducing lipid peroxidation markers and supporting GSH homeostasis, essential for counteracting oxidative damage (Figure 10).

These findings suggest that the tested isoflavonoids possess significant mitochondrial-protective and antioxidant properties, likely through membrane stabilization and free radical scavenging mechanisms. Their ability to counteract t-BuOOH-induced oxidative stress highlights their potential as neuroprotective agents, further supporting their pharmacological relevance in mitochondrial dysfunction-related disorders.

### 2.6. Effects on Isolated Rat Brain Microsomes

Microsomes are vesicle-like fragments formed in vitro from the endoplasmic reticulum during cellular fragmentation. While they do not naturally exist in healthy, living cells, they can be isolated through differential centrifugation and serve as experimental models for studying drug metabolism, lipid peroxidation, and the enzymatic activity of the cytochrome P450 system. Due to their ability to retain key enzymes involved in Phase I and Phase II biotransformation, microsomes are widely utilized for evaluating metabolic stability and oxidative stress responses [48].

**Figure 11:**
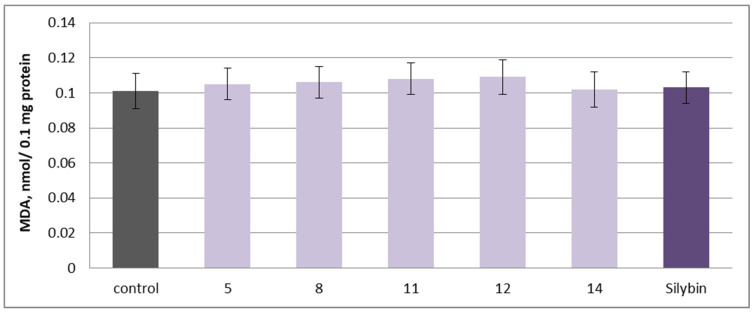
Effects of **5**, **8**, **11**, **12**, **14** and silybin, applied individually at a concentration of 100 µM, on MDA production in isolated rat brain microsomes.

To assess the pro-oxidant potential, the isolated **5**, **8**, **11**, **12**, **14** substances and silybin were applied individually at 100 µM in isolated rat brain microsomes. No statistically significant increase in malondialdehyde (MDA) production, a key biomarker of lipid peroxidation, was observed, indicating that the tested substances do not induce oxidative stress in this model (Figure 11).

Reactive oxygen species (ROS), including superoxide anion (O_2_^−^) and hydrogen peroxide (H_2_O_2_), are continuously generated as byproducts of aerobic respiration. While they play essential roles in cellular signaling, excessive accumulation leads to oxidative damage, particularly affecting lipids, proteins, and DNA. Unsaturated fatty acids in cell membranes are highly susceptible to ROS attack, leading to membrane dysfunction and the formation of toxic lipid peroxides. These peroxides can further react with iron ions, generating epoxide–peroxide radicals, which amplify the lipid peroxidation cascade. The final breakdown products of this process, such as MDA and 4-hydroxynonenal, are highly reactive and contribute to cellular toxicity and neurodegeneration [47].

**Figure 12:**
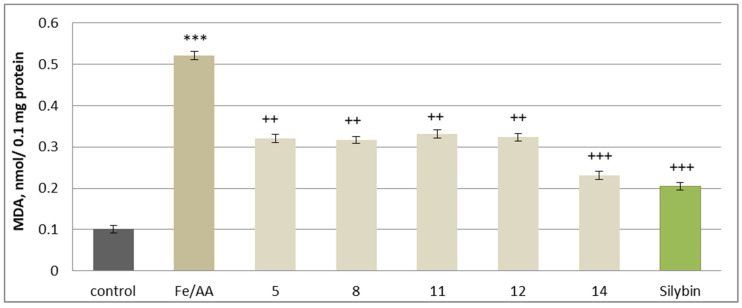
Effects of **5**, **8**, **11**, **12**, **14**, and silybin at a concentration of 50 µM on non-enzymatically induced lipid peroxidation, measured by MDA production in isolated rat brain microsomes. *** *p* < 0.001 vs. control (untreated microsomes); ^++^ *p* < 0.01; ^+++^ *p* < 0.001 vs. control (iron/ascorbate only).

A widely accepted non-enzymatic lipid peroxidation model involves iron/ascorbate-induced oxidative stress, which mimics conditions of excessive ROS production. In this system, the iron/ascorbate combination significantly increased MDA production by 416%, confirming its role as a strong oxidative stress inducer. The antioxidant capacity of the tested substances was evaluated in this oxidative stress model. When co-administered at 50 µM, the investigated **5**, **8**, **11**, **12**, **14** substances and silybin demonstrated significant inhibition of MDA formation, suggesting a protective effect against iron/ascorbate-induced lipid peroxidation. The strongest antioxidant activity was observed with maackiain and silybin, which reduced MDA production by 56% and 61%, respectively. Additional protective effects were noted with cajanin (**12**) and onogenin (**11**), reducing MDA levels by 38% and 36%, respectively. Substances **5** and **8** also displayed notable antioxidant activity, reducing MDA levels by 38% and 39%, compared to the oxidative stress control (Figure 12).

The results indicate that the tested isoflavonoids effectively counteract oxidative stress at the microsomal level, likely through membrane stabilization and direct antioxidant mechanisms. Their ability to suppress lipid peroxidation and maintain cellular integrity further supports their therapeutic potential in conditions associated with oxidative damage and neurodegeneration.

### 2.7. Molecular Docking Results

**Figure 13:**
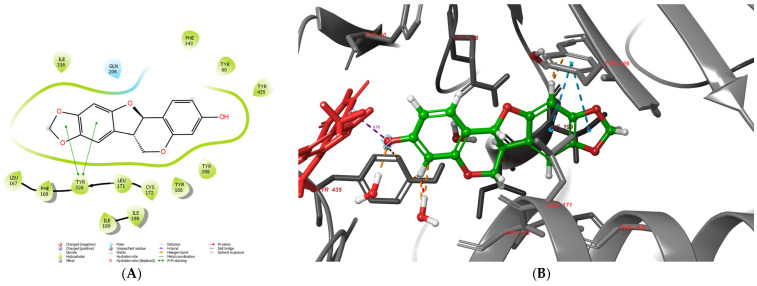
Active conformation of maackiain (**14**) in monoamine oxidase type B—2D (**A**) and 3D (**B**) representation. The active ligand was represented by green sticks; the protein with grey ribbons; the co-enzyme FAD was depicted in red. The dotted lines indicate stabilizing interactions in the active site: orange for water-mediated interactions, blue for polar interactions, and purple for the measured distance to FAD (3.72 Å).

The docking scores demonstrated that several ligand isomers of odoratin (**2**), fujikinetin (**4**), 6, 4′-dimethoxy-7, 2′-dihidroxy (**7**) are hypothetically better MAO-B inhibitors compared to the molecule of the standard selegiline when the IFD scores are observed. Interestingly, some of these ligands were tested as inactive MAO-B inhibitors during the in vitro studies. However, the main drawback of the docking simulations is the inability of the scoring algorithms to differentiate true active molecules from decoys [49]. Notably, the docking simulations successfully discriminated several true actives from inactive compounds. None of the ligands demonstrate docking values comparable to the irreversable MAO-B inhibitor, safinamide (IFD—16.14 kcal/mol). Overall, the applied docking protocols, which included the utilizaiton of three modules, Glide XP, MM/GBSA and IFD, identified 10 active hit molecules, which could be used as MAO-B inhibitors. The experimental testing validated the hypothetical data, showing that five of these ligands possess good to moderate blocking capacities towards the enzyme. To evaluate the active conformation of the most active MAO-B inhibitor, maackiain, both 2D and 3D panels of the intermolecular interactions are represented in Figure 13.

The active site of monoamine oxidase type B is divided into three sections (domains)—entrance cavity, substrate pocked and “aromatic cage”. The most favorable interactions with these domains are hydrophobic in nature [50]. Notably, the aromatic cage comprises two active amino acids, Tyr435 and Tyr398, together with the co-enzyme FAD. Maackiain is well suited in the active pocket of MAO-B. The hydroxyl moiety is faced towards the FAD of the aromatic cage. Moreover, Tyr435 and Tyr398 stabilize the potent MAO-B inhibitor through hydrophobic interactions. The distance between the hydroxyl moiety and N5 of FAD is 3.72 Å, which increases the reliability of the docking protocol, as reported elsewhere [51]. Maackiain was also involved in a hydrophobic interaction with Ile199, which acts as a “gate” between the two active cavities of the enzyme. Moreover, ligands which interact with Ile199 enhance MAO-B selectivity [52]. Two stable p-p bonds were formed between the 1,3-benzodioxole fragment of the active ligand and Tyr326. The distances of the bonds were 4.77Å and 5.05 Å. The active amino acid Tyr326 is involved in the formation of a loop, which separates the entrance pocket from the substrate cavity. Furthermore, increased MAO-B inhibiting effects were displayed when Tyr326 was involved in the stabilization with the examined ligands [53]. The active amino acids Tyr60, Leu167, Phe168, Leu171, Cys172, Tyr188, Ile198, Ile199 and Ile316 were involved in hydrophobic interactions with maackiain, which further stabilized the protein–ligand complex.

## 3. Discussion

### 3.1. Occurrence of the Isolated Phytochemicals

Among the 14 structures isolated from *A. aitosensis*, several stand out for their rarity and represent the first reports of these metabolites in the *Astragalus* genus. These include 3′-methoxydaidzein (**3**), previously identified only in unrelated genera, such as *Sophora tonkinensis* [54], *Pueraria lobata* [55], *Dalbergia odorifera* and *D. louvelii* [32,56]; fujikinetin (**4**), earlier reported in *Dalbergia frutescens* [57], *Bowdichia virgilioides* [34], and *Cyclopia intermedia* (Honeybush tea) [58]; and 6,4′-dimethoxy-7,2′-dihydroxyisoflavone (**7**), previously found only in *Dalea spinosa* [32]. Likewise, sayanedine (**8**) appears to be a highly uncommon metabolite, with prior reports limited to *Pisum sativum* [37,59]. Onogenin (**11**) is another compound rarely detected, having been reported only in a few Fabaceae species such as *Ononis spinosa*, *O. arvensis* [42,43], and *Ulex europaeus* [59,60]. Given that these metabolites have also been reported in distantly related genera across the Fabaceae family, their occurrence in *A. aitosensis*, a phylogenetically and evolutionary old species, may reflect the retention of early-evolved metabolic routes that are no longer active or have been significantly altered in more recently diverged *Astragalus* species. Investigating whether these phytochemicals occur in other members of the genus could offer important conclusions regarding the evolutionary dynamics of isoflavonoid biosynthesis and help clarify whether their presence in *A. aitosensis* represents a unique adaptation or a shared, but underrecognized, phytochemical feature across the genus. It is also noteworthy that some of these metabolites, such as onogenin and formononetin, function as phytoalexins or phytohormones, playing critical roles in plant defense and stress response [11,61,62,63]. Their presence may reflect adaptation to the rocky, eroded soils and rough microclimate that define the natural habitat of *A. aitosensis*.

From a phytochemical perspective, these findings expand the structural diversity of *Astragalus* isoflavonoids beyond the well-established metabolites such as calycosin, formononetin, and ononin [22,23,64,65,66,67,68]. Our results also support the idea that endemic and taxonomically neglected species can harbor structurally rare or pharmacologically potent molecules. Importantly, ten of the isolated substances displayed favorable docking scores, and five showed selective MAO-B inhibition, with maackiain exhibiting the most potent activity, approaching that of the standard inhibitor selegiline.

### 3.2. Pharmacological Evaluation

The pharmacological assessment of the isolated isoflavonoids revealed promising neuroprotective potential, particularly through selective inhibition of monoamine oxidase-B (MAO-B). Among the **14** tested phytochemicals, **5** exhibited notable MAO-B inhibitory activity, with maackiain emerging as the most potent, achieving 45% inhibition at 10 µM, approaching the effect of the reference drug selegiline (55%). Cajanin (**12**), onogenin (**11**), afrormosin (**5**), and sayanedine (**8**) also demonstrated measurable inhibition, ranging from 25% to 35%, while the remaining compounds showed no significant effect.

In parallel, these active substances consistently display neuroprotective effects in oxidative stress models using isolated rat brain synaptosomes, mitochondria, and microsomes. Under exposure to 6-OHDA or t-BuOOH, the active phytochemicals notably preserved intracellular glutathione (GSH) levels and reduced malondialdehyde (MDA) production, markers of oxidative damage. Maackiain (**14**) again showed the strongest activity, reducing MDA levels by over 50% and maintaining GSH concentrations close to control levels. These effects were further supported by lipid peroxidation assays, where all five active substances reduced iron/ascorbate-induced oxidative damage, with maackiain and cajanin (**12**) showing inhibition levels of 56% and 38%, respectively.

Together, these results represent a group of structurally related isoflavonoids with dual pharmacological properties, inhibiting MAO-B and mitigating oxidative stress, which may have therapeutic relevance in neurodegenerative disorders such as Parkinson’s disease.

### 3.3. Structure–Activity Relationship

A comparative evaluation of the isolated molecules revealed a distinct structure–activity relationship that correlates with their selective MAO-B inhibition and associated neuroprotective effects. Although several of the isoflavonoids share a similar core scaffold—either a chromen-4-one (isoflavone) or fused tricyclic (pterocarpan/neoflavonoid)—only some demonstrated significant MAO-B inhibition. This contradiction proves that small differences in substitution, ring fusion and electronic properties substantially affect bioactivity. Among the active metabolites, maackiain (**14**), onogenin (**11**), cajanin (**12**), afrormosin (**5**) and sayanedine (**8**) displayed selective MAO-B inhibition, with maackiain reducing enzyme activity by 45%, closely approaching the reference inhibitor selegiline (55%). In contrast, structurally related analogues, such as formononetin (**10**), 3′-methoxydaidzein (**3**), trifoliol (**13**) and pseudobaptigenin (**9**), showed little or no activity. The most active is maackiain, which features a pterocarpan scaffold with a rigid tricyclic ring system and a 1,3-benzodioxole moiety. These structural elements promote optimal orientation for π–π stacking and hydrophobic interactions with critical MAO-B residues, including Tyr398, Tyr435, and Ile199, located within the enzyme’s “aromatic cage.” The C7-OH group, positioned near the FAD cofactor, appears essential for stabilizing the ligand through hydrogen bonding and proximity effects, as confirmed by docking simulations and favorable MM/GBSA scores. Qsar analysis (hydrophobicity/TPSA/H-bonding descriptors plus 3D interaction fingerprints) ranked maackiain as the top MAO-B ligand; the benzodioxole-driven p-stacking surface and a single C7-H bond donor were the highest-weighted features, explaining their potency and MAO-B selectivity. This QSAR prioritization mirrors the experimental profile (45% inhibition, approaching selegiline) and aligns with the literature data, which supports maackiain as a strong MAO-B inhibitor. Compounds like onogenin and cajanin, although based on a simpler isoflavone skeleton, also contain C7-OH and C4′-OH/OCH_3_ groups, facilitating polar interactions and moderate hydrophobic contacts. These features may explain their intermediate activity profiles (30–35% inhibition). Sayanedine, bearing C3′-OH and C7-OCH_3_, displayed modest activity, suggesting that hydroxylation at the B ring supports MAO-B binding, even when C7 is methoxylated. In contrast, formononetin and 3′-methoxydaidzein, despite having the same isoflavone core, lack either critical hydroxyls or have substituents (e.g., methoxy at C3′) that may reduce hydrogen bonding potential or introduce steric hindrance. Similarly, trifoliol (**13**) from the group of coumestans and pseudobaptigenin (**9**), which share a pterocarpan-like scaffold with maackiain, remain inactive, likely due to suboptimal ring orientation or the absence of key pharmacophoric groups. Collectively, these results suggest that free hydroxyl groups at C7 and/or C4′, a hydrophobic methylenedioxy system, and a rigid three-ring core (as in pterocarpans) contribute strongly to MAO-B inhibition. Inactive compounds often exhibited unfavorable substitution patterns, lower planarity, or lacked key interactions with the aromatic cage.

## 4. Materials and Methods

### 4.1. Plant Material

Aerial parts of native *A. aitosensis* were collected under the authorization of the Bulgarian Ministry of Environment and Water (License № 949/18 August 2022) from their natural habitat near Aytos, Bulgaria (42.72° N, 27.27° E). These samples were authenticated and deposited in the herbarium of the Bulgarian Academy of Sciences, “Index Herbariorum” (BAS-SOM), with voucher number 178665.

### 4.2. General Experimental Procedures

1D and 2D NMR (COSY, HSQC, HMBC) experiments were conducted using a Bruker AVII+ 600 spectrometer (Bruker, Karlsruhe, Germany) at a proton NMR frequency of 600.13 MHz in CD_3_OD (99.5%, Deutero GmbH, Kastellaun, Germany) and DMSO (99.9%, Deutero GmbH, Kastellaun, Germany) and a carbon NMR frequency of 150.90 MHz with TMS as the internal standard. NMR spectra were processed and analyzed using TopSpin software version 4.4.1 (Bruker Biospin GmbH, Ettlingen, Germany). An LC-HRESI-MS analysis of pure compounds was carried out using a Thermo Scientific Q Exactive Plus Quadrupole-Orbitrap mass spectrometer (Thermo Fisher Scientific, Bremen, Germany) in ultra-high-resolution mode (70,000 at *m*/*z* 200), coupled with a UPLC Dionex Ultimate 3000 RSLC system (Thermo Fisher Scientific, Waltham, MA, USA). An RP-18 Kinetex column (2.1 × 100 mm, 2.6 µM, Phenomenex, Torrance, CA, USA) was used with a gradient elution of MS-grade solvents A (0.1% FA in H_2_O) and B (0.1% FA in MeCN) as follows: 0 min—5% B, 0.5 min—25% B, 5.5 min—35% B, 8.5 min—95% B, 9.5 min—95% B. The mobile phase flow rate was 0.3 mL/min, and the column temperature was maintained at 40 °C. Injection volume was 2.5 µL. Data were acquired in both positive and negative ionization modes. The full MS scan lasted 9.5 min (0.5–10.0 min run time) with a resolution of 70,000, AGC target of 3 × 10^6^, maximum IT of 100 ms, and scan range of 150–1500 *m*/*z*. MS/MS scans were performed at a resolution of 17,500, AGC target of 1 × 10^5^, maximum IT of 50 ms, scan range of 200–2000 *m*/*z*, isolation window of 2.0 *m*/*z*, and stepped (N)CE at 10, 30, and 60. The ionization parameters were as follows: nitrogen dry gas flow 8.0 L/min, capillary temperature 320 °C, source temperature 320 °C, sheath gas flow 36 AU, auxiliary flow 11 AU, source voltage 3.5 kV, and capillary voltage 320 V. Data acquisition and processing were performed using Thermo Xcalibur 2.2 software (Thermo Fisher Scientific, Waltham, MA, USA). All solvents, including MeOH and CH_2_Cl_2_, were at least of analytical grade (Fischer Chemicals, Loughborough, UK). Milli-Q water (Millipore, Bedford, MA, USA) was filtered through a 0.22 µM membrane before use. UV spectra were measured on a LIBRA S70 UV/vis spectrophotometer covering 190–1100 nm (Libra, Cambridge, UK). Column chromatography (CC) was performed at atmospheric pressure using a glass column (40 × 3 cm) packed with Polyamide CC6 (Supelco, Darmstadt, Germany). Semi-preparative high-performance liquid chromatography (semi-prep HPLC) was conducted using a Young Lin 9100 system (Young Lin Instrument Co., Ltd., Anyang, Republic of Korea), equipped with a vacuum degasser, YL9110 quaternary pump, YL9160 photodiode array (DAD) detector, manual injector (7725), and YL Clarity software 4.0.3.876.

### 4.3. Extraction and Isolation

The aerial parts of *A. aitosensis* (2 kg) were extracted with 15 L of 80% MeOH. The obtained total extract (184.9 g) was subjected to liquid–liquid extraction, dissolved in 2 L acidified H_2_O (HCl, pH < 3) and subsequently extracted three times with 2 L CH_2_Cl_2_. The pH of remaining aqueous phase was adjusted to pH > 9 (NH_3_aq) and extracted again three times with 2 L CH_2_Cl_2_ (fraction A). The solvent was evaporated, and fraction A dissolved in CH_2_Cl_2_/MeOH (1:1) was subjected to further purification with Polyamide CC6 (CC). Elution was carried out using CH_2_Cl_2_/MeOH mixtures of increasing polarity, each in 500 mL volumes, in the following sequence: CH_2_Cl_2_, CH_2_Cl_2_/MeOH (9:1), CH_2_Cl_2_/MeOH (8:2), CH_2_Cl_2_/MeOH (7:3), CH_2_Cl_2_/MeOH (6:4), CH_2_Cl_2_/MeOH (2:8), and finally MeOH. This procedure yielded seven fractions, labeled A1 to A7. Based on HPLC profile of the fractions, A3, A4 and A5 were selected for continuing the purification and isolation.

### 4.4. Pharmacology Procedures

The pharmacological evaluation of the isolated phytochemicals was conducted through in vitro biochemical assays and ex vivo experiment using subcellular fractions from rat brain tissue. The experiments adhered to Ordinance No. 15 for the protection and humane treatment of experimental animals (State Gazette No. 17, 2006) and the European Directive for Experimental Animal Work, under permission No. 273 (valid until 20 July 2025) issued by the Bulgarian Food Safety Agency (BFSA). Male Wistar rats (1.5–2 years old) were obtained from the National Breeding Center, Bulgarian Academy of Sciences, Slivnitsa, Bulgaria, and housed under standard laboratory conditions before experimental procedures. Brain synaptosomes, mitochondria, and microsomes were isolated through differential centrifugation, following the methods of Taupin et al. [69] and Sims and Anderson using a Percoll gradient [70]. The protein content of each fraction was determined using the Lowry method [71]. Synaptosomes were incubated with 150 µM 6-hydroxydopamine [72], mitochondria were treated with 75 µM tert-butyl hydroperoxide (t-BuOOH) [73], and microsomes were isolated following the protocol of Ravindranath and Anandatheerthavarada [74] via ultracentrifugation at 100,000× *g* for 1 h. The viability of synaptosomes following incubation with the test compounds was evaluated using the MTT assay [75]. Determination of reduced glutathione (GSH) levels in synaptosomes was performed using Ellman’s reagent (DTNB) [76]. After incubation, synaptosomes were centrifuged at 400× *g* for 3 min, treated with 5% trichloroacetic acid (TCA), and stored at −20 °C before analysis. For mitochondrial GSH quantification, a 0.04% DTNB solution was added before spectrophotometric measurement at 412 nm [77]. Lipid peroxidation was assessed by measuring malondialdehyde (MDA) levels using the thiobarbituric acid (TBA) assay [77]. For mitochondrial lipid peroxidation, 0.3 mL of 0.2% thiobarbituric acid and 0.25 mL of 0.05 M sulfuric acid were added, and the mixture was boiled for 30 min. After cooling, 0.4 mL of n-butanol was added, followed by centrifugation at 3500× *g* for 10 min. The absorbance of MDA was recorded spectrophotometrically at 532 nm. Lipid peroxidation in microsomes was induced using 20 μM FeSO_4_ and 0.5 mM ascorbic acid, and MDA levels were quantified at 535 nm [48]. The inhibitory activity of the isolated structures against human recombinant MAO-A and MAO-B enzymes was analyzed using a fluorometric Amplex UltraRed assay [78], employing tyramine hydrochloride as the substrate. Data were statistically processed using MEDCALC software 23.3.7, with significance levels set at *p* < 0.05, *p* < 0.01, and *p* < 0.001, while the MAO-A/B activity results were analyzed using GraphPad Prism 5.0.

### 4.5. Molecular Docking

The X-ray structure of MAO-B was retrieved from the protein data bank (PDB) with co-crystallized safinamide and a resolution of 1.60 Å [79]. The protein was fully prepared for the docking studies by utilizing the Maestro module—Protein Preparation [80]. The latter was used for the generation of hydrogen bonds, the states at physiological pH values, minimization of the final protein structure by using OPLS4 force field. The grid box was generated with Receptor Grid Generation included in Maestro by forming a box around the co-crystallized ligand. The title structures were prepared by applying LigPrep—neutralizing charged groups, energy minimization, generating ionization states at pH 7.0 ± 0.4. For the docking calculations, Glide with XP scoring was used. Furthermore, Induced-Fit Docking (IFD) was used as it observes the active amino acids as fully flexible and provides improved robustness to the theoretical results. The recalculations of the binding free energies were also presented by applying the MM/GBSA (Molecular Mechanics-Generalized Born Surface Area) module.

## 5. Conclusions

This work represents the first comprehensive phytochemical and pharmacological evaluation of *A. aitosensis*, an endemic species with limited prior chemical study. Through a simple but targeted extraction approach using pH-controlled conditions, we were able to selectively isolate a set of ionizable natural molecules. This method proved both practical and effective, leading to the isolation and structural characterization of two previously unreported molecules: isomer of odoratin (**2**) and 6-hydroxy-3-(2-hydroxy-4-methoxyphenyl)-7-methoxy-4*H*-1-benzopyran-4-one (**6**), and some rare metabolites, which had not been documented previously in the genus *Astragalus*, including 3′-methoxydaidzein (**3**), fujikinetin (**4**), sayanedine (**8**), and 6,4′-dimethoxy-7,2′-dihydroxyisoflavone (**7**). From a phytochemical point of view, these findings expand the known diversity of isoflavonoids in *Astragalus*, beyond the well-documented molecules like calycosin and formononetin. The presence of metabolites typically found in distantly related Fabaceae genera suggests that *A. aitosensis* may retain generic biosynthetic bases. This supports the idea that underexplored endemic species can serve as valuable sources of structurally unique and biologically relevant compounds.

On the pharmacological side, based on the few literature data, the phytochemicals were not well studied but showed prominent pharmacological activity. Our study further investigated and supported their therapeutical potential with new data. Five susbtances, maackiain (**14**), cajanin (**12**), onogenin (**11**), afrormosin (**5**), and sayanedine (**8**), showed clear biological activity, particularly through selective inhibition of monoamine oxidase-B (MAO-B), an enzyme linked to neurodegenerative conditions. Maackiain (**14**) demonstrated the strongest activity, approaching that of the standard drug selegiline. These active metabolites of *A. aitosensis* also performed well in oxidative stress models, helping to maintain glutathione levels and reduce lipid peroxidation in vitro.

The structure–activity relationship (SAR) based on the conducted docking analysis helped clarify why only certain structures were active. Specific features, such as free hydroxyl groups at C7 and/or C4′, methoxylation at defined positions, and a rigid fused-ring system, were associated with higher MAO-B inhibition and neuroprotective effects. The substances lacking these features, despite structural similarity, showed no significant activity. Taken together, this study demonstrates that a simple extraction approach can effectively uncover rare and active metabolites from neglected plant species. It also shows that traditional phytochemical work, when paired with pharmacological testing and SAR analysis, remains a strong strategy for identifying promising lead structures in natural product research.

This research highlights the imperative of conserving biodiversity to protect potential medicinal sources that could significantly impact global healthcare. Continued exploration and research into other unexplored species within the *Astragalus* genus and other similar genera will undoubtedly yield additional valuable insights and novel therapeutic agents.

## Figures and Tables

**Table 1:** ^1^H and ^13^C NMR assignments (600 MHz) for structures 2 and 6 (*δ* (ppm), multiplicity (*J* in Hz)).

	2		6	
Position	δ_C_	δ_H_	δ_C_	δ_H_
2	152.7, s	8.36, s	152.5, s	8.23, s
3	n.o.	–	125.1, s	–
4	174.4, s		174.1, s	–
5	107.3, d	7.38, s	104.5, d	7.39, s
6	153.8, s	–	n.o.	–
7	145.7, s	–	147.5, s	–
8	100.1, d	7.15, s	n.o.	6.86, s
9	150.8, s	–	152.6, s	–
10	117.5, s	–	n.o.	
1′	123.1, s	–	122.9, s	–
2′	113.0, d	7.16, d (2.0 Hz)	146.1, s	–
3′	147.4, s	–	119.8, d	6.94, s
4′	146.5, s	–	147.5, s	–
5′	114.9, d	6.80, d (8.2 Hz)	116.6, d	7.03, s
6′	121.3, d	6.99, dd (8.18, 2.05 Hz)	112.0, d	6.94, s
-OCH_3_-	55.95, q (C-6)	3.91, s	55.75, q (C-7)	3.86, s
-OCH_3_-	55.48, q (C-3′)	3.78, s	55.67, q (C-4′)	3.78, s

s: singlet; d: doublet; q: quaternary; n.o.: not observed.

## Data Availability

The data presented in this study are available on request from the corresponding author.

## Supplementary Materials

The following supporting information can be downloaded at: https://www.mdpi.com/article/10.3390/molecules30204069/s1.
