# Supplementary material for: Phenolic Derivatives of Astragalus aitosensis with Selective MAO-B Inhibition and Mitochondrial Protection
Source: Molecules. 2025 Oct 13;30(20):4069. doi: 10.3390/molecules30204069 (PMC12566051; doi:10.3390/molecules30204069)
Supplement: Supplementary file 1 [file molecules-30-04069-s001.zip › molecules-3867073-supplementary.pdf]

SUPPLEMENTARY MATERIALS

Phenolic Derivatives of *Astragalus Aitosensis* with Selective MAO-B Inhibition and Mitochondrial Protection

Preslav Enchev[1], Magdalena Kondeva-Burdina[2], Emilio Mateev[3], Iliana Ionkova[1], Yancho Zarev[1]

Affiliations

[1] Department of Pharmacognosy, Faculty of Pharmacy, Medical University of Sofia, 2 Dunav Str., 1000 Sofia, Bulgaria; p.enchev@pharmfac.mu-sofia.bg (P.E.); ionkova@pharmfac.mu-sofia.bg (I.I.); yzarev@pharmfac.mu-sofia.bg (Y.Z)

[2] Department of Pharmacology, pharmacotherapy and toxicology, Faculty of Pharmacy, Medical University of Sofia, 2 Dunav Str., 1000 Sofia, Bulgaria; mkondeva@pharmfac.mu-sofia.bg (M.K.)

[3] Department of Pharmaceutical Chemistry, Faculty of Pharmacy, Medical University of Sofia, 2 Dunav Str., 1000 Sofia, Bulgaria; e.mateev@pharmfac.mu-sofia.bg (E.M.)

Corresponding author: Yancho Zarev, yzarev@pharmfac.mu-sofia.bg

Table of content:

1. Figure S1. Chromatogram and MS spectrum of methyl ferulate (**1**) $C_{11}H_{12}O_4$
2. Figure S2. Chromatogram and MS spectrum of 3'-methoxydaidzein (**3**)
3. Figure S3. Chromatogram and MS spectrum of fujikinetin (**4**)
4. Figure S4. Chromatogram and MS spectrum of afrormosin (**5**)
5. Figure S5. Chromatogram and MS spectrum of 6,4'-dimethoxy-7,2'-dihydroxy isoflavone (**7**)
6. Figure S6. Chromatogram and MS spectrum of sayanedine (**8**)
7. Figure S7. Chromatogram and MS spectrum of psuedobaptigenin (**9**)
8. Figure S8. Chromatogram and MS spectrum of formononetin (**10**)
9. Figure S9. Chromatogram and MS spectrum of onogenin (**1**1)
10. Figure S10 Chromatogram and MS spectrum of cajanin (**12**)
11. Figure S11. Chromatogram and MS spectrum of trifoliol (**13**)
12. Figure S12. Chromatogram and MS spectrum of maackiain (**14**)
13. Figure S13. Chromatogram and MS spectrum of odoratin isomer (**2**)
14. Figure S14. ^{1}H NMR spectrum of odoratin isomer (**2**)
15. Figure S14a. ^{1}H NMR spectrum of odoratin isomer (**2**)
16. Figure S14b. ^{1}H NMR spectrum of odoratin isomer (**2**)
17. Figure S15. COSY spectrum of odoratin isomer (**2**)
18. Figure S16. HSQC spectrum of odoratin isomer (**2**)
19. Figure S17. HMBC spectrum of odoratin isomer (**2**)
20. Figure S18 Chromatogram and MS spectrum of 6-hydroxy-3-(2-hydroxy-4-methoxyphenyl)-7-methoxy-4H-1-benzopyran-4-one (**6**)
21. Figure S19. ^{1}H NMR spectrum of 6-hydroxy-3-(2-hydroxy-4-methoxyphenyl)-7-methoxy-4H-1-benzopyran-4-one (**6**)
22. Figure S19a. ^{1}H NMR spectrum of 6-hydroxy-3-(2-hydroxy-4-methoxyphenyl)-7-methoxy-4H-1-benzopyran-4-one (**6**)
23. Figure S19b. ^{1}H NMR spectrum of 6-hydroxy-3-(2-hydroxy-4-methoxyphenyl)-7-methoxy-4H-1-benzopyran-4-one (**6**)
24. Figure S20. ^{13}C NMR spectrum of 6-hydroxy-3-(2-hydroxy-4-methoxyphenyl)-7-methoxy-4H-1-benzopyran-4-one (**6**)
25. Figure S21. COSY spectrum of 6-hydroxy-3-(2-hydroxy-4-methoxyphenyl)-7-methoxy-4H-1-benzopyran-4-one (**6**)
26. Figure S22. HSQC spectrum of 6-hydroxy-3-(2-hydroxy-4-methoxyphenyl)-7-methoxy-4H-1-benzopyran-4-one (**6**)
27. Figure S23. HMBC spectrum of 6-hydroxy-3-(2-hydroxy-4-methoxyphenyl)-7-methoxy-4H-1-benzopyran-4-one (**6**)

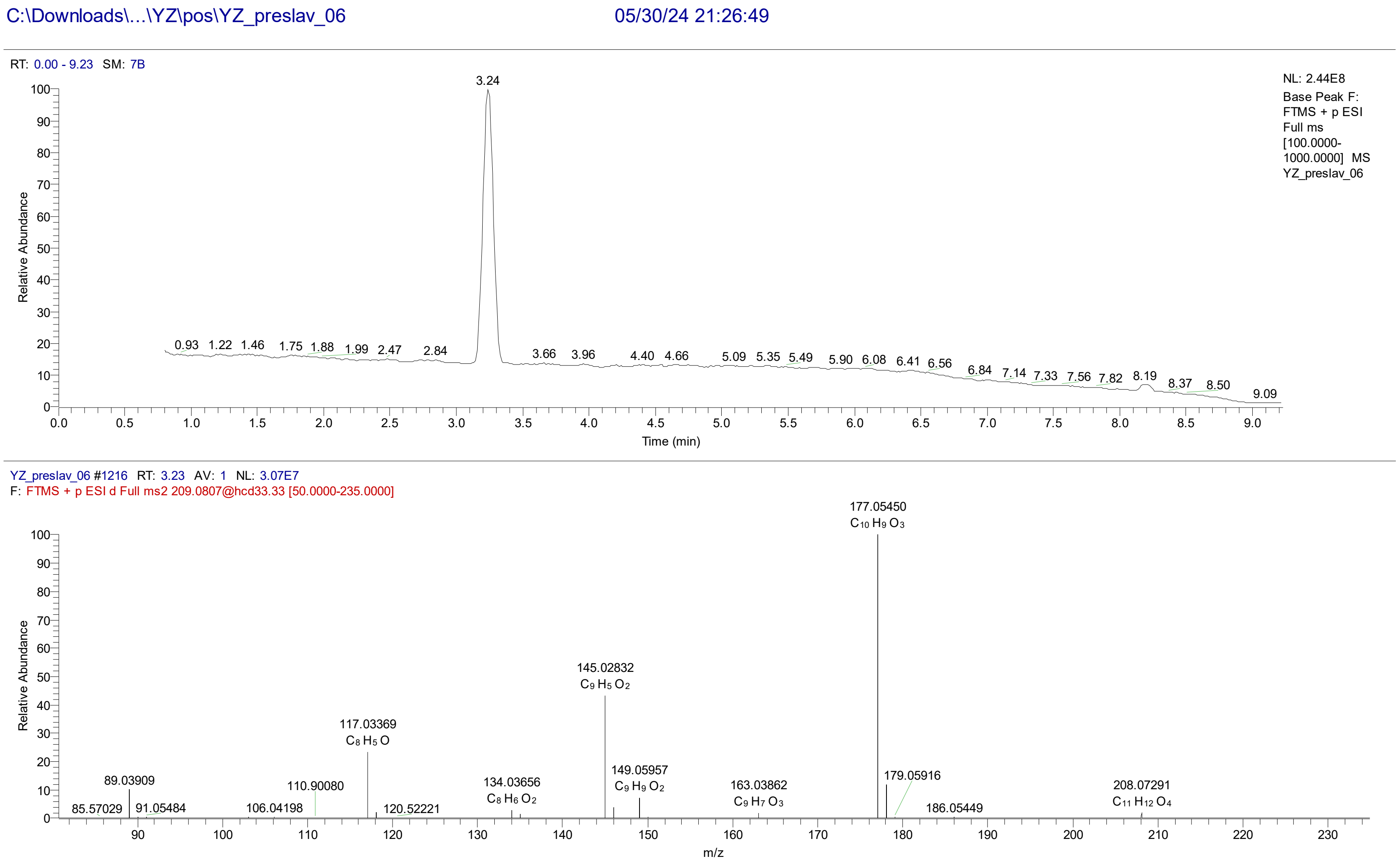

Figure S1. Chromatogram and MS spectrum of methyl ferulate (**1**) $C_{11}H_{12}O_4$.

Compound (**1**) was isolated as white amorphous powder (1.3 mg) observed as protonated molecular ion with *m/z* 209.0806 corresponding to molecular formula $C_{11}H_{13}O_4^+$ (calcd.= 209.0808) and retention time of 3.24′.

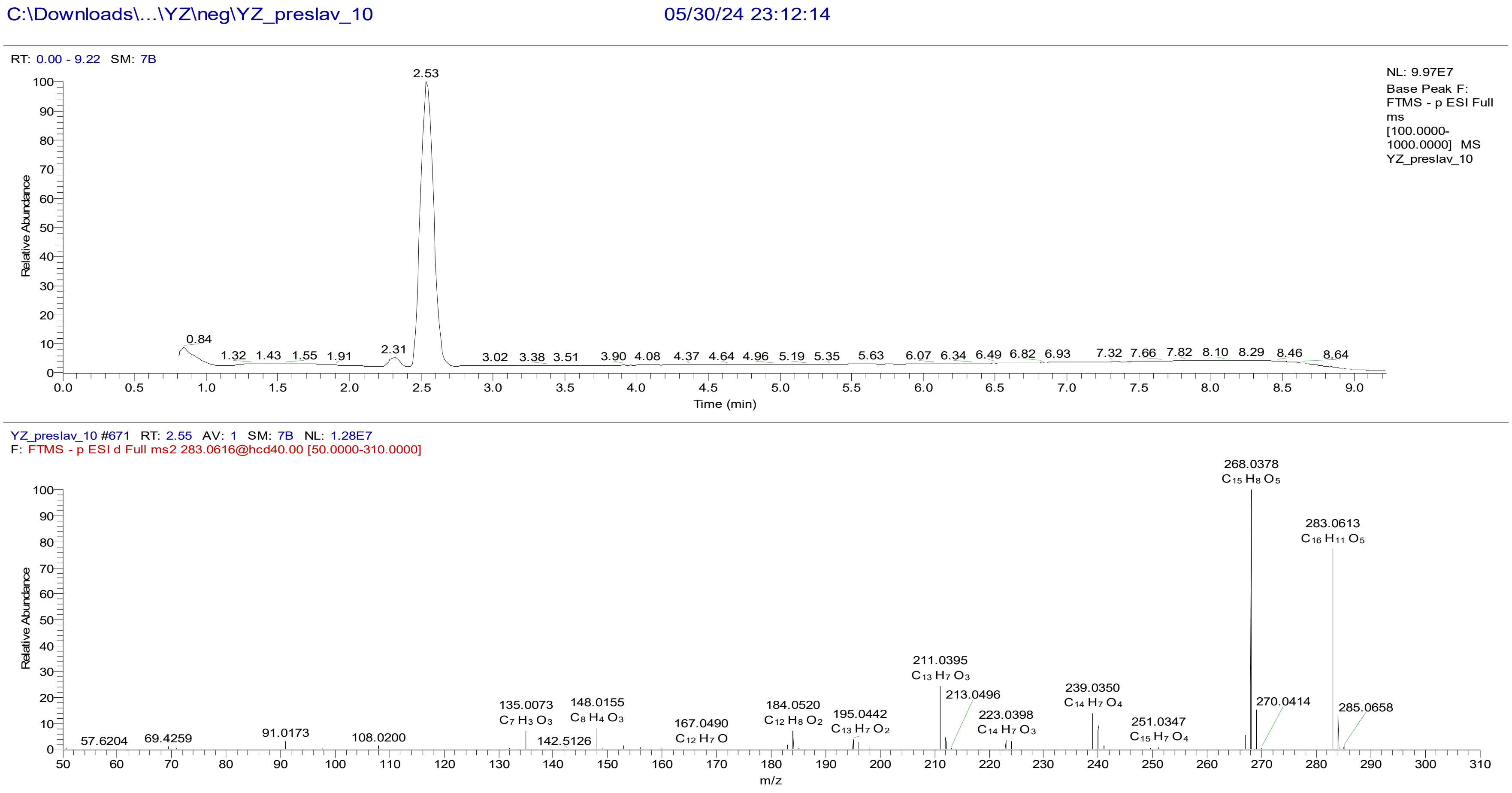

Figure S2. Chromatogram and MS spectrum of 3′-methoxydaidzein (**3**).

Compound (**3**) was isolated as white amorphous powder (1.2 mg) observed as deprotonated molecular ion with *m/z* 283.0613 corresponding to molecular formula $C_{16}H_{11}O_5^-$ (calcd.= 283.0601) and retention time of 2.53′.

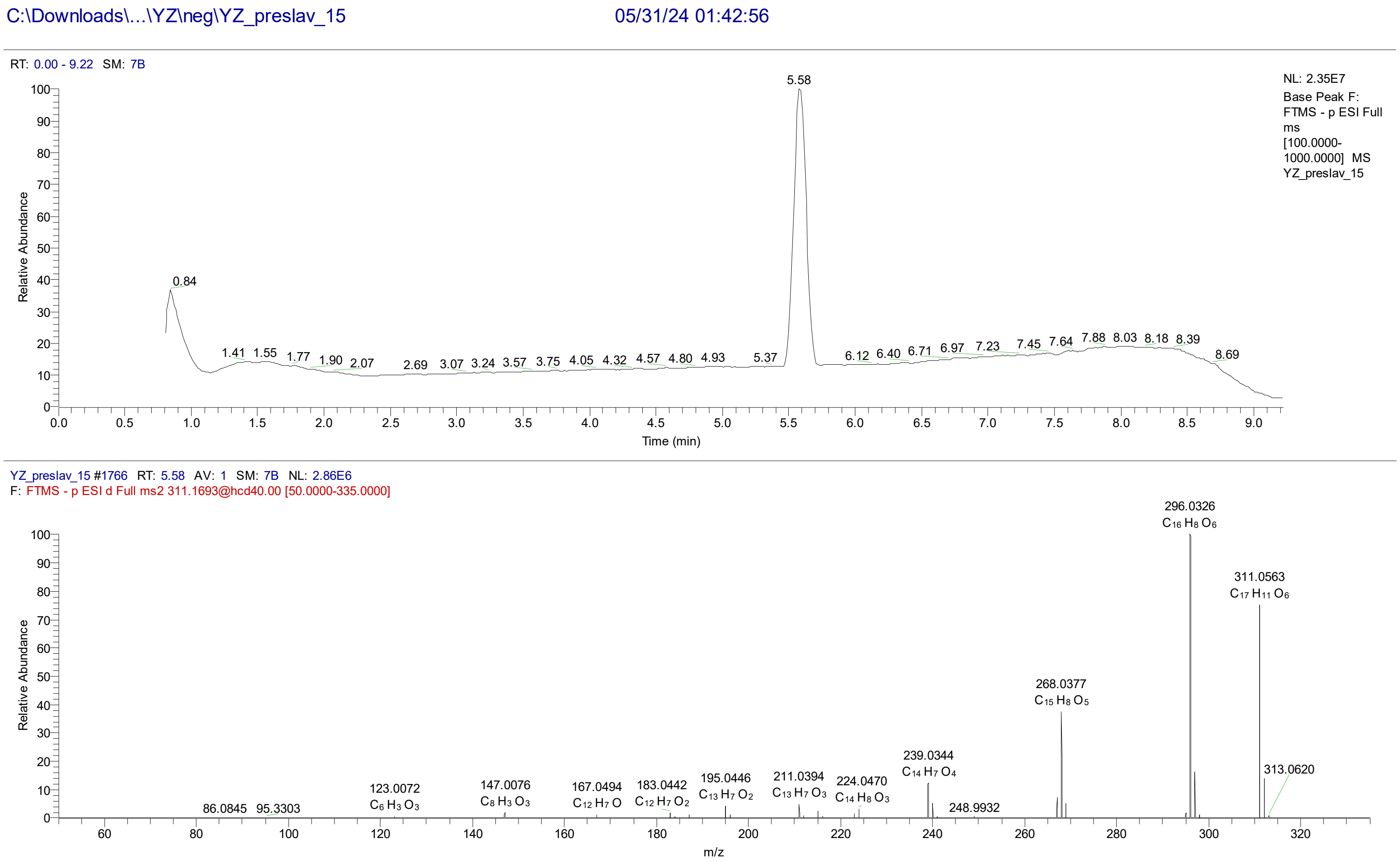

Figure S3. Chromatogram and MS spectrum of fujikinetin (**4**).

Compound (**4**) was isolated as white amorphous powder (1.6 mg) observed as deprotonated molecular ion with *m/z* 311.0563 corresponding to molecular formula $C_{17}H_{11}O_6^-$ (calcd.=311.0550) and retention time of 5.58′.

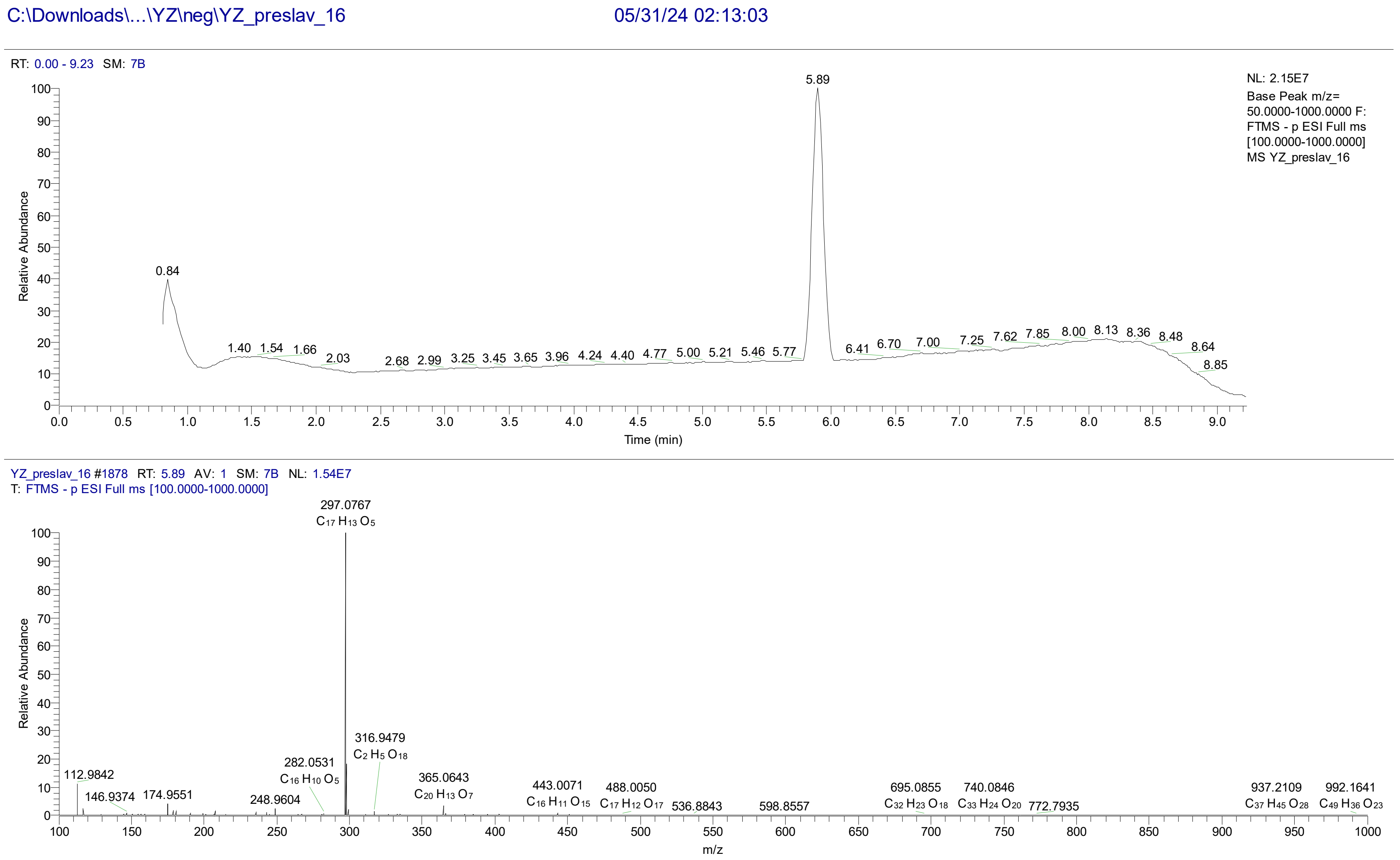

Figure S4. Chromatogram and MS spectrum of afrormosin (**5**).

Compound (**5**) was isolated as white amorphous powder (1.5 mg) observed as molecular ion with *m/z* 297.0767 corresponding to molecular formula $C_{17}H_{13}O_5^-$ (calcd.=297.0757) and retention time of 5.89′.

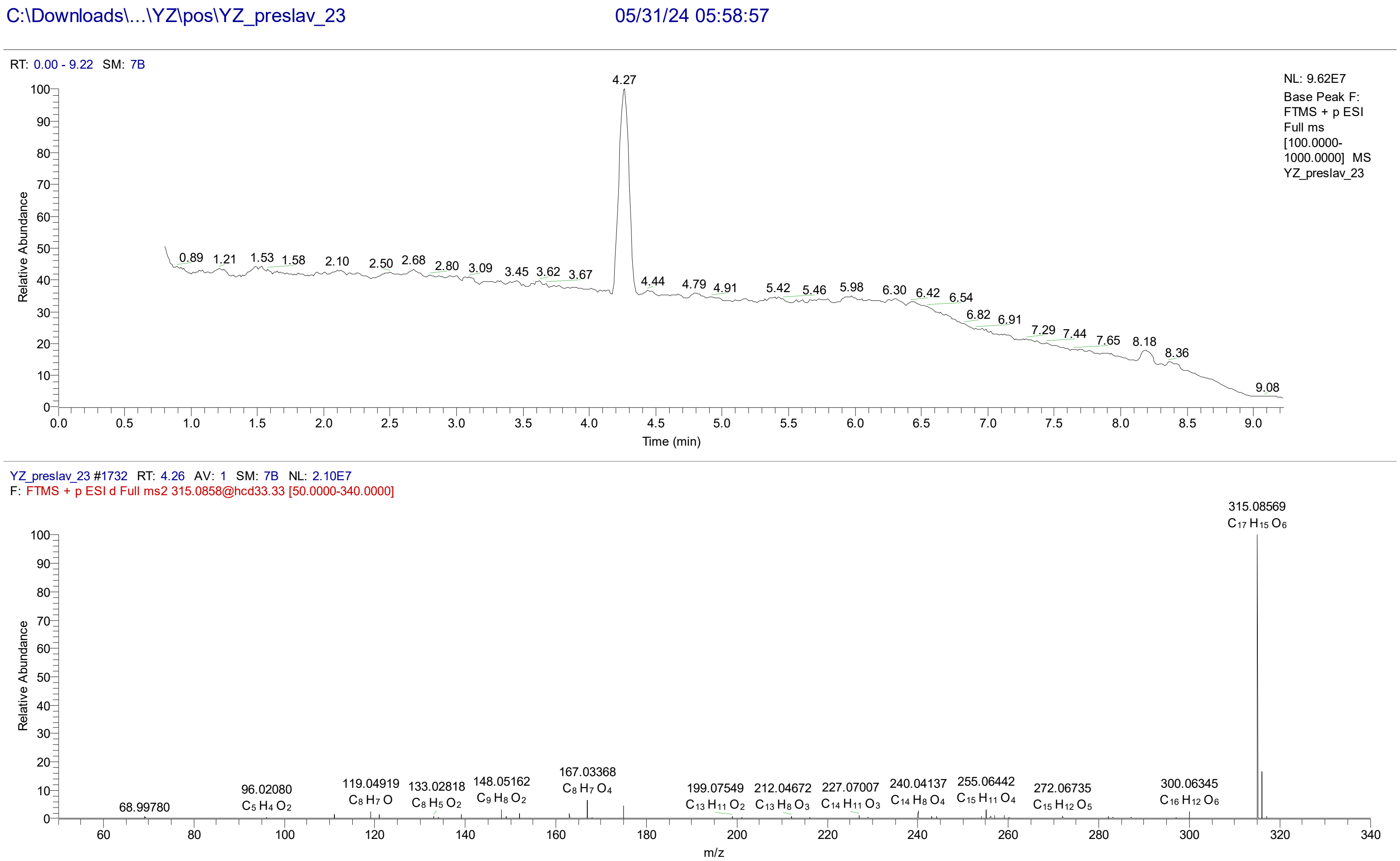

Figure S5 Chromatogram and MS spectrum of 6, 4′-dimethoxy-7, 2′-dihydroxy isoflavone (**7**).

Compound (**7**) was isolated as white amorphous powder (1.0 mg) and observed as protonated molecular ion with *m/z* 315.0857 corresponding to molecular formula $C_{17}H_{15}O_6^+$ (calcd. 315.0863) and retention time of 4.27′.

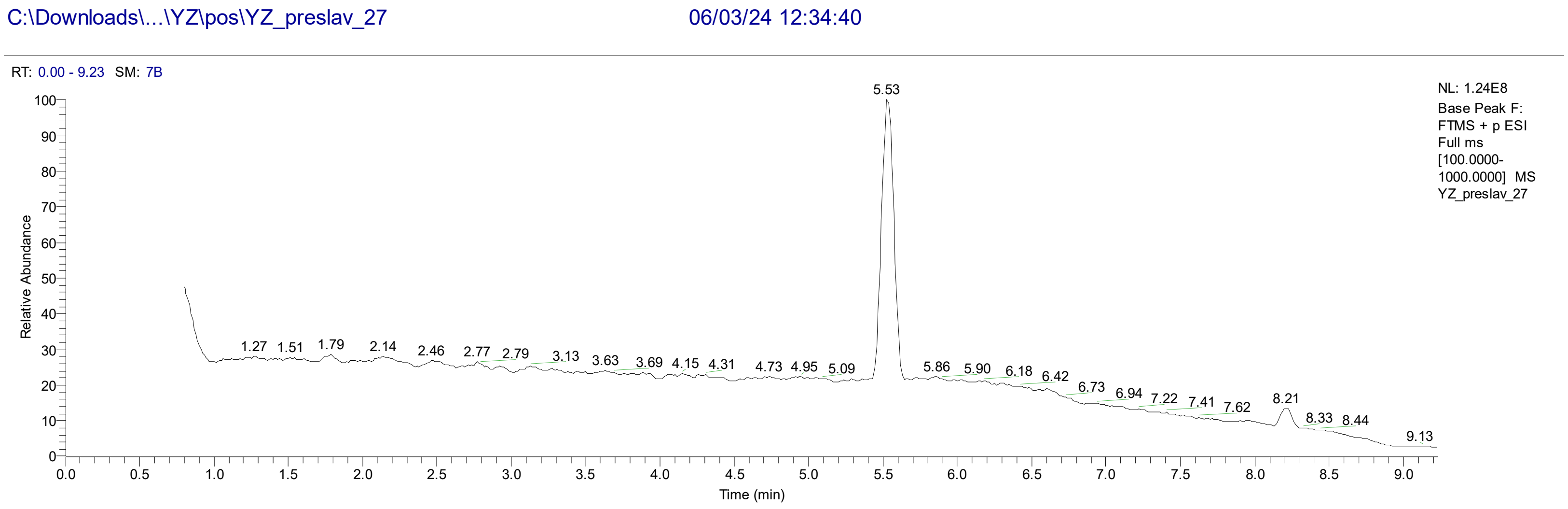

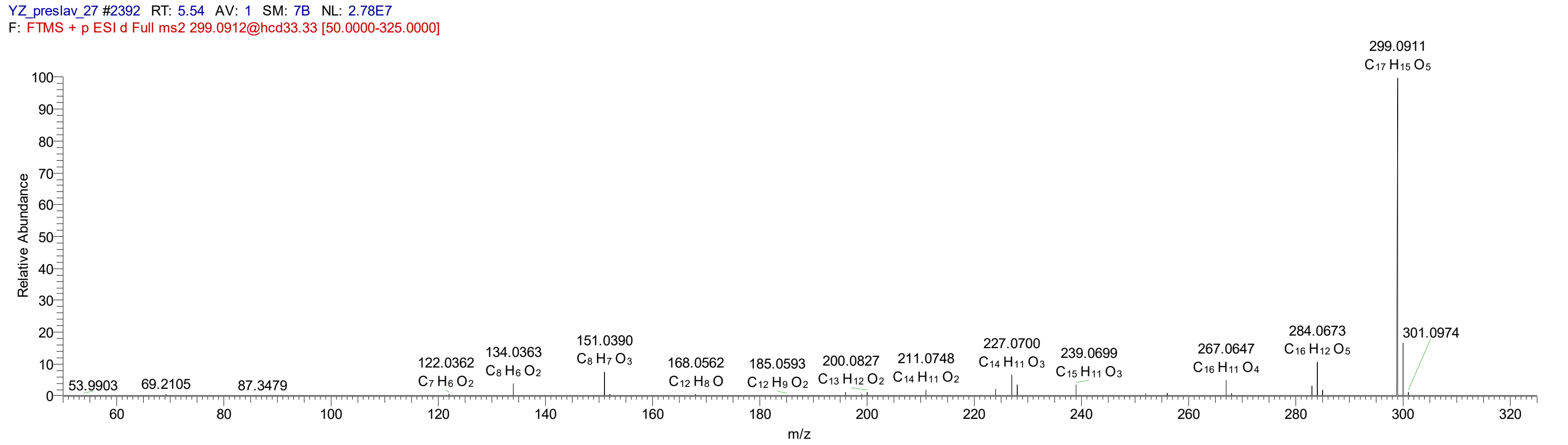

Figure S6. Chromatogram and MS spectrum of sayanedine (**8**)

Compound (**8**) was isolated as white amorphous powder (2.0 mg) observed as protonated molecule ion at *m/z* 299.0911 corresponding to molecular formula $C_{17}H_{14}O_6^+$ (calcd. 299.0914) and retention time of 5.53′.

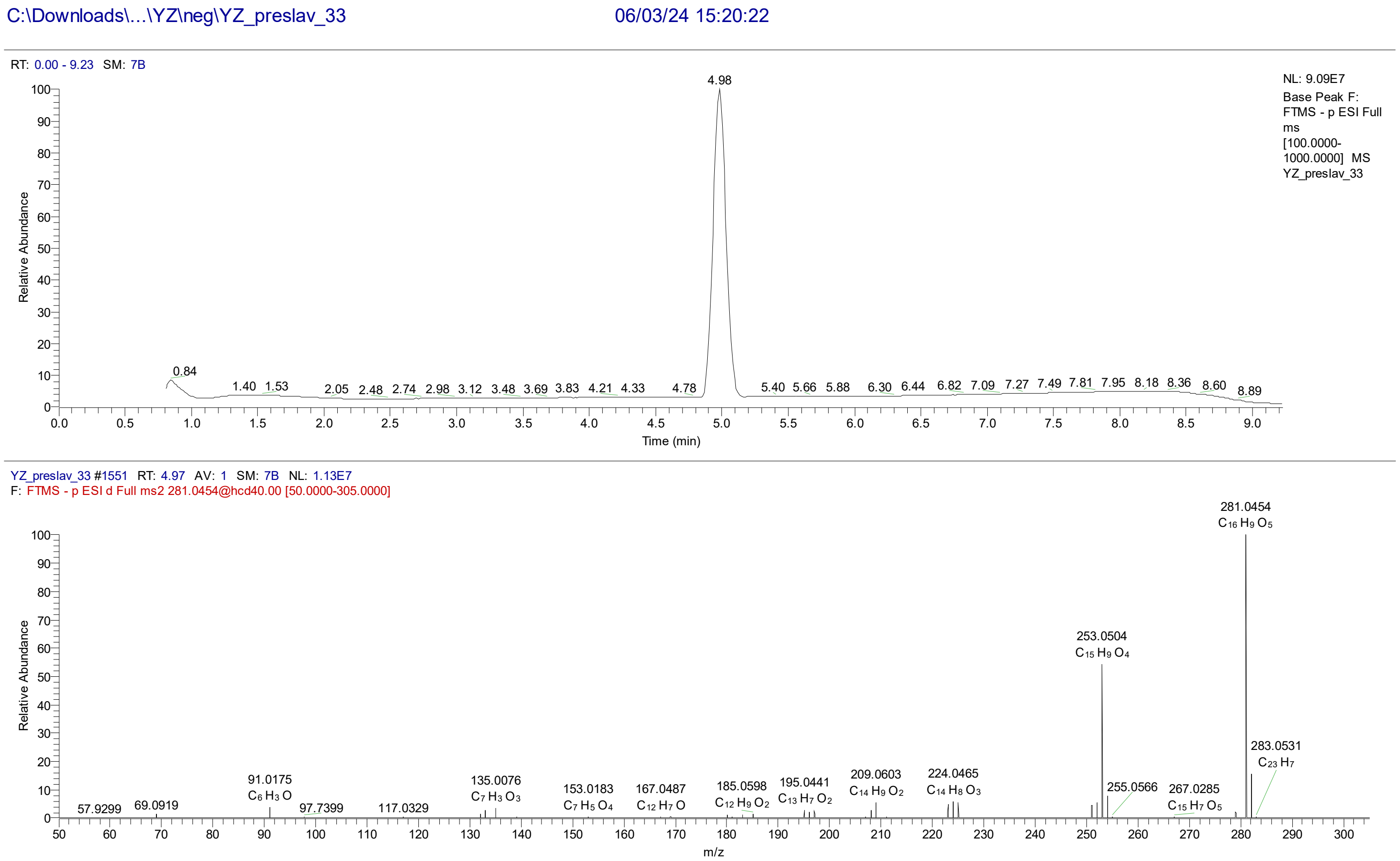

Figure S7. Chromatogram and MS spectrum of psuedobaptigenin (**9**).

Compound (**9**) was isolated as white amorphous powder (0.7 mg) and observed as deprotonated molecular ion with *m/z* 281.0454 corresponding to molecular formula$(C_{16}H_9O_5)^-$ (calcd. 281.0444) and retention time of 4.98′.

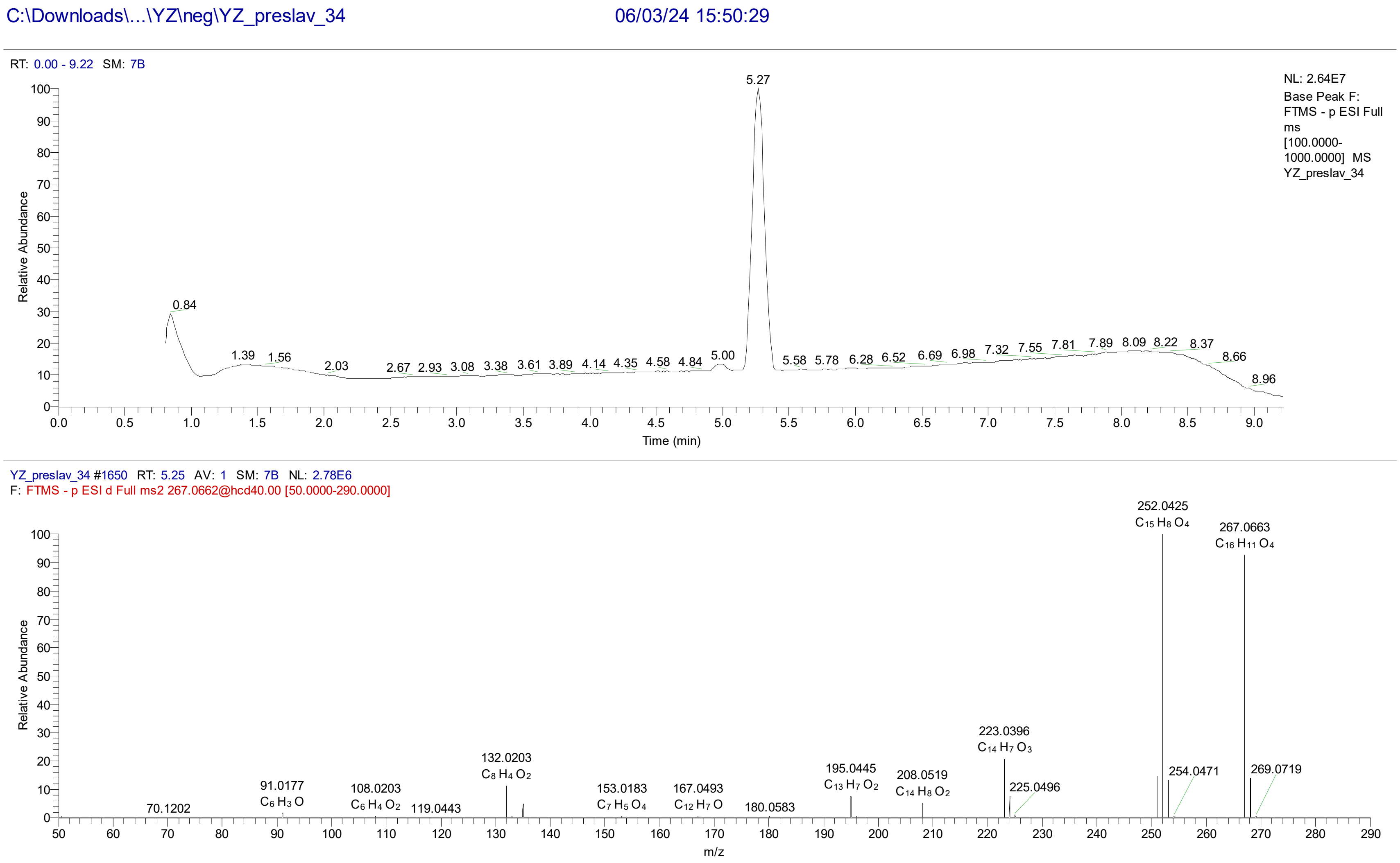

Figure S8. Chromatogram and MS spectrum of formononetin (**10**)

Compound (**10**) was isolated as white amorphous powder (0.6 mg) observed at 5.27′ as deprotonated molecular ion with *m/z* 267.0663 corresponding to macular formula $C_{16}H_{11}O_4^-$ (calcd. 267.0652).

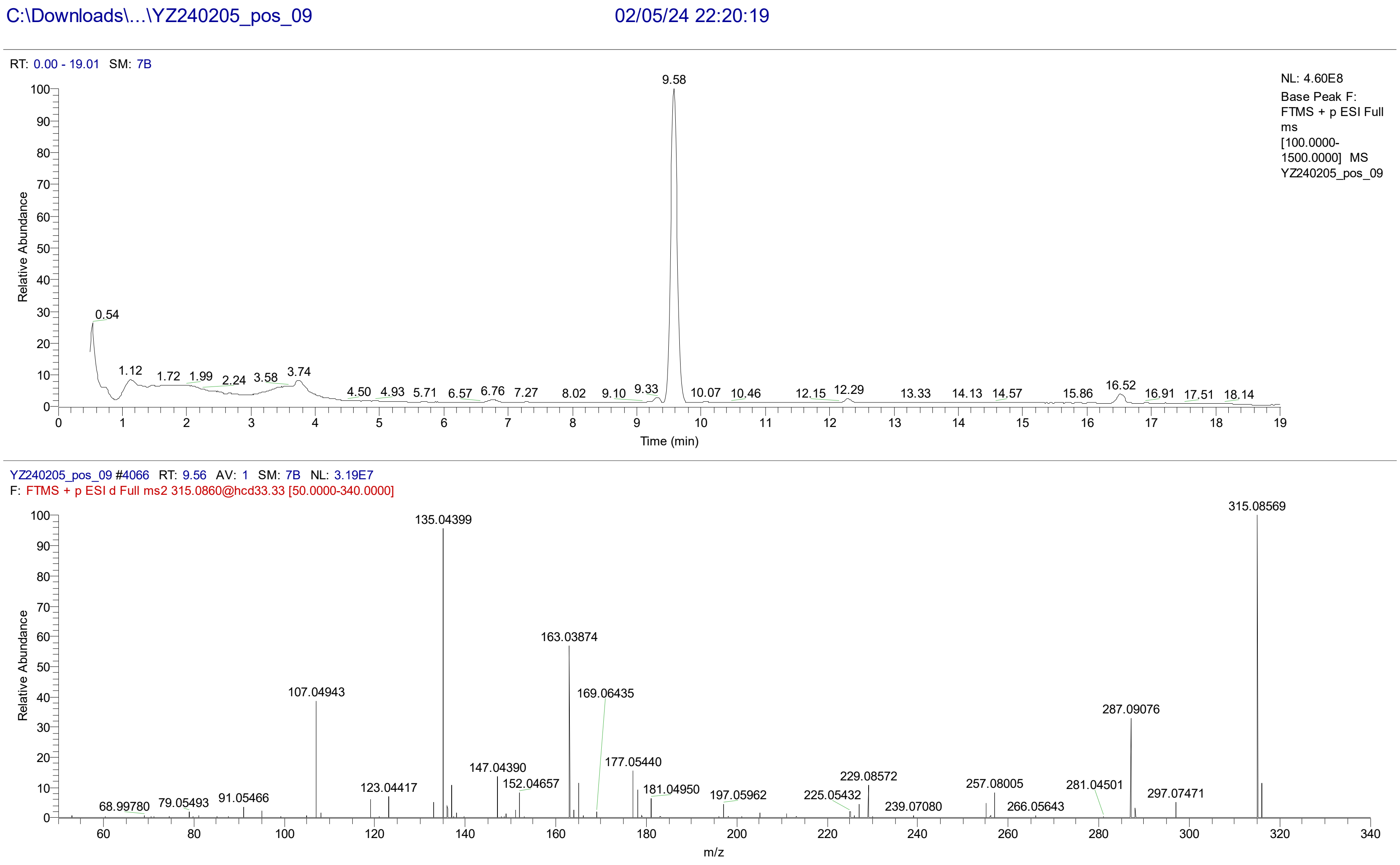

Figure S9. Chromatogram and MS spectrum of onogenin (**11**)

Compound **11** was isolated as white amorphous powder (1.6 mg) and observed as protonated molecular ion with *m/z* 315.0856 corresponding to molecular formula$(C_{17}H_{15}O_6)^+$ (calcd. 315.0863) and retention time 9.58′.

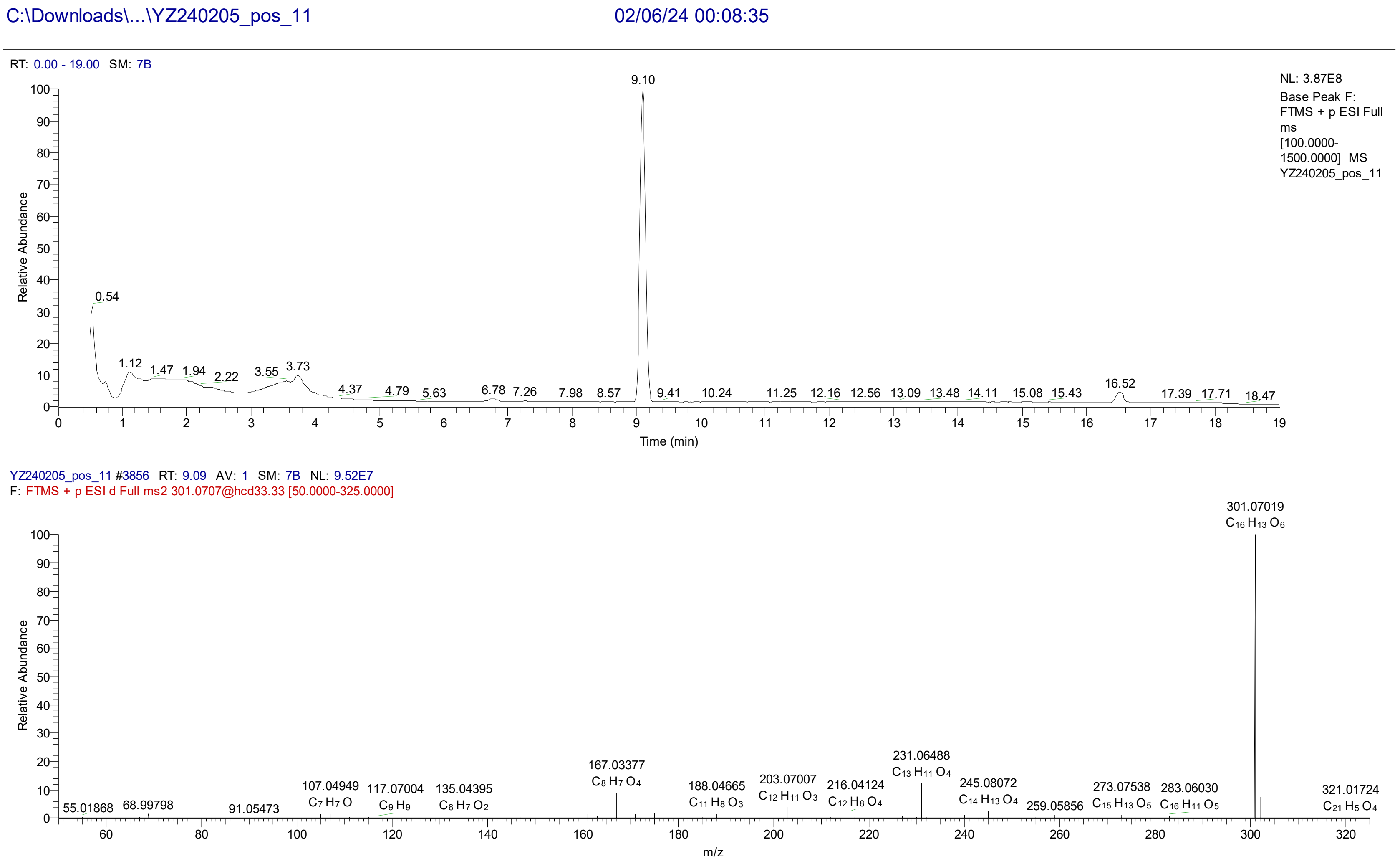

Figure S10. Chromatogram and MS spectrum of cajanin (**12**).

Compound **12** was isolated as white amorphous powder (0.9 mg) and observed as protonated molecular ion with *m/z* 301.0702 corresponding to molecular formula$(C_{16}H_{13}O_6)^+$ (calcd. 301.0707) and retention time 9.10′.

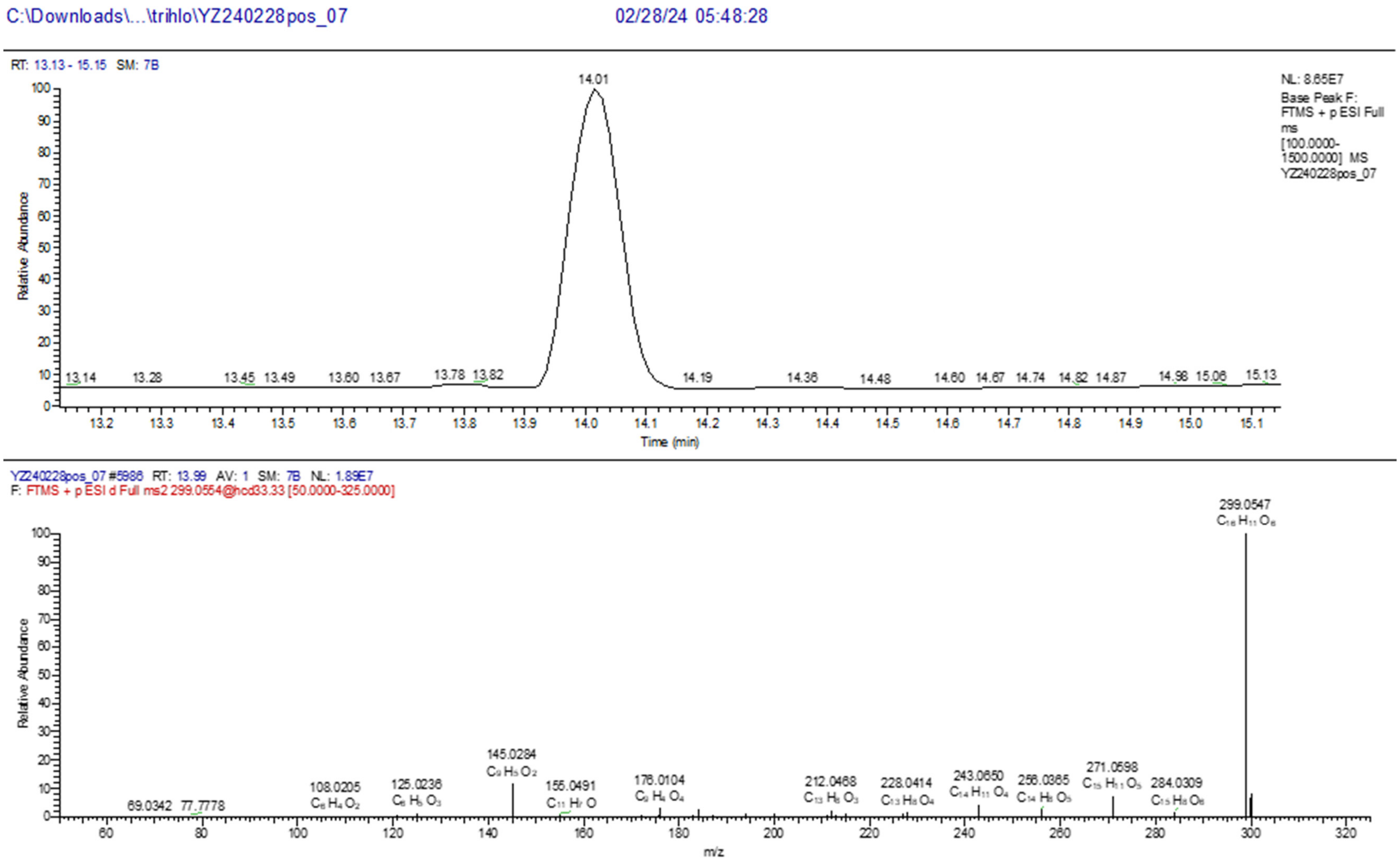

Figure S11. Chromatogram and MS spectrum of trifoliol (**13**)

Compound **13** was isolated as white amorphous powder (1.3 mg) and observed as protonated molecular ion with *m/z* 299.0549 corresponding to molecular formula$C_{16}H_{11}O_6^+$ (calcd. 299.0550) and retention time 14.02′.

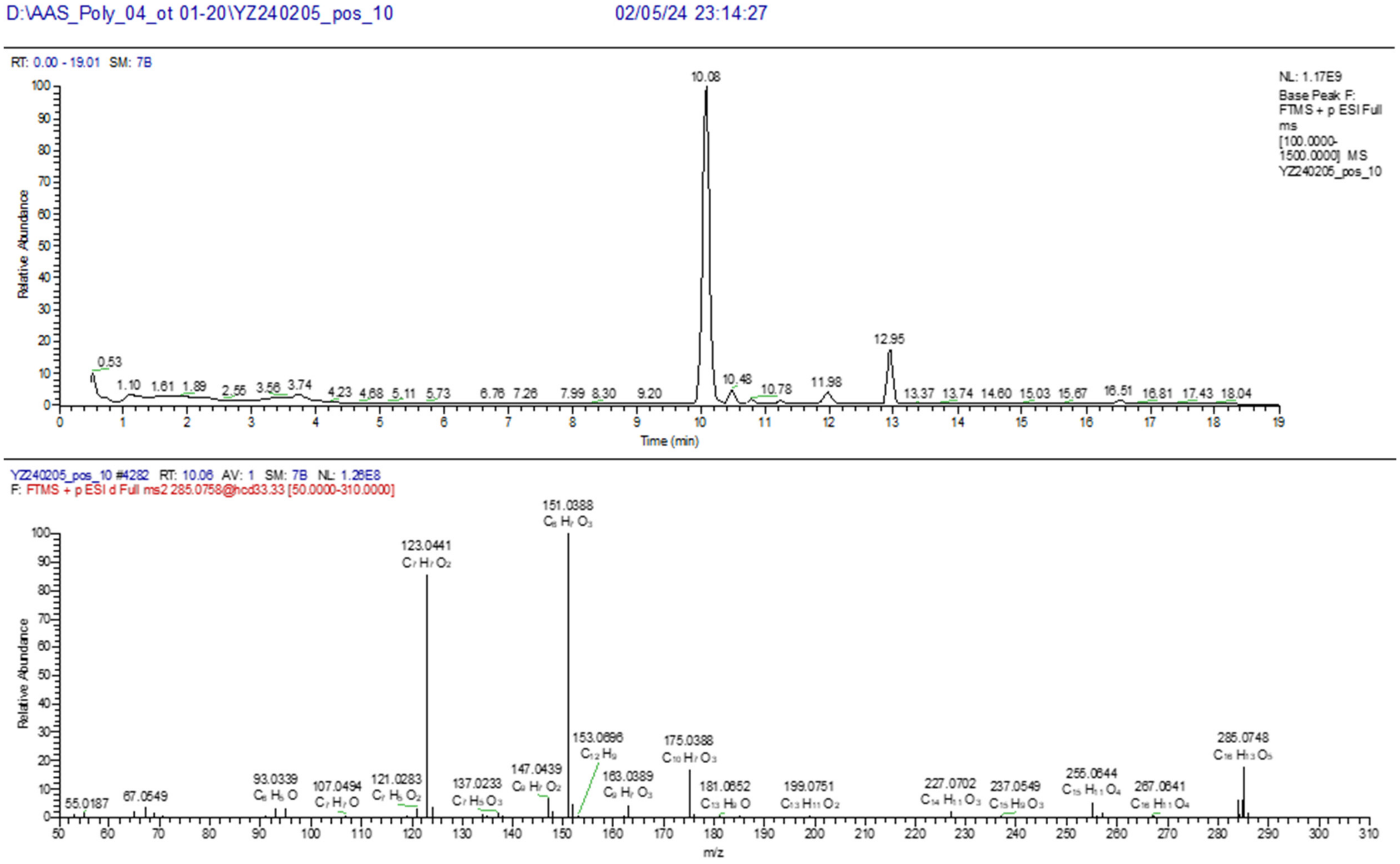

Figure S12. Chromatogram and MS spectrum of maackiain (**14**)

Compound **14** was isolated as white amorphous powder (48.6 mg) and observed as protonated molecular ion with *m/z* 285.0753 corresponding to molecular formula $C_{16}H_{13}O_5^+$ (calcd. 285.0757) and retention time 14.02′.

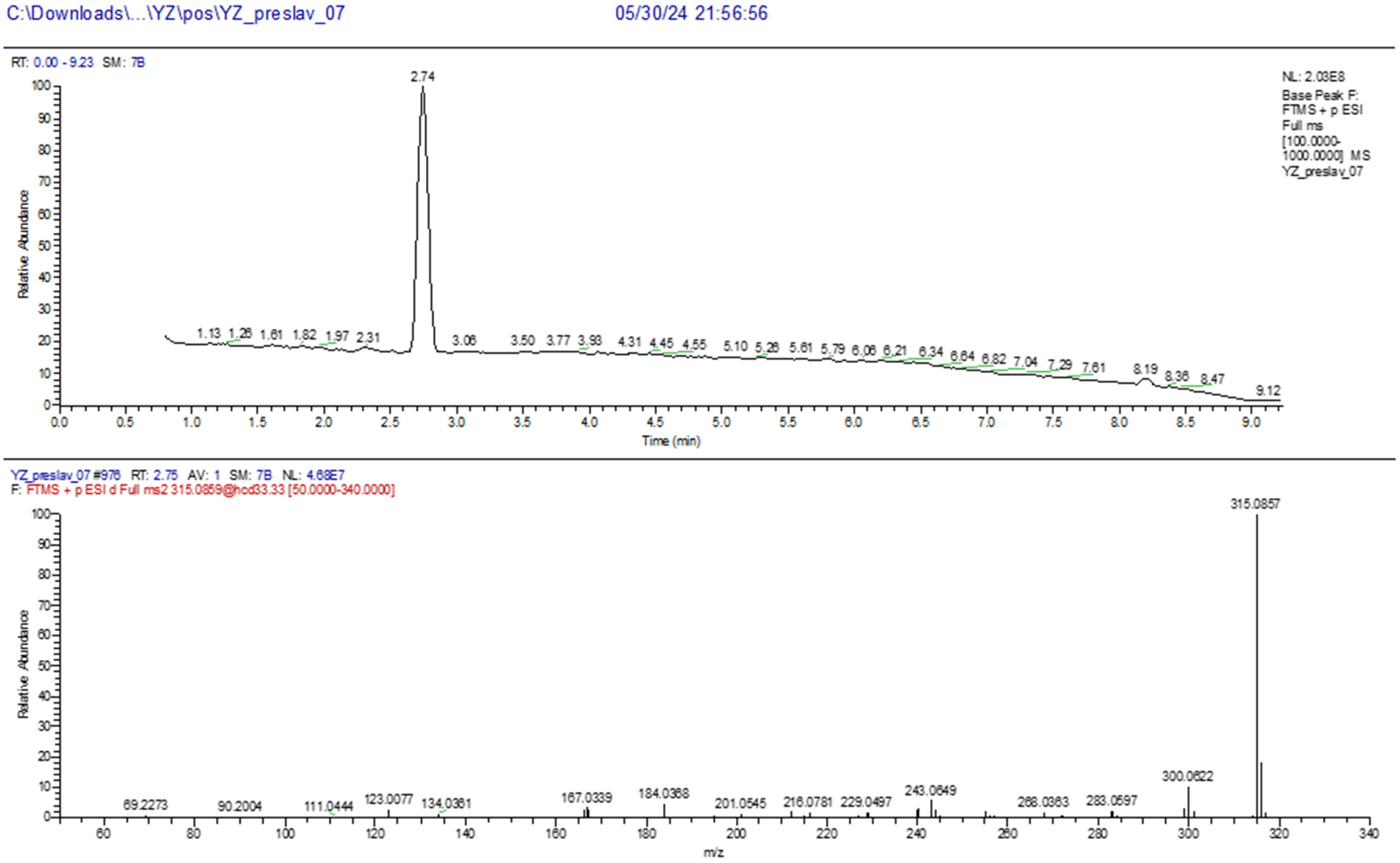

Figure S13. Chromatogram and MS spectrum of odoratin isomer (**2**)

Compound **2** was isolated as white amorphous powder (0.7 mg) and observed as protonated molecular ion with *m/z* 315.0858 corresponding to molecular formula$C_{17}H_{15}O_6^+$ (calcd. 315.0863) and retention time 2.74′.

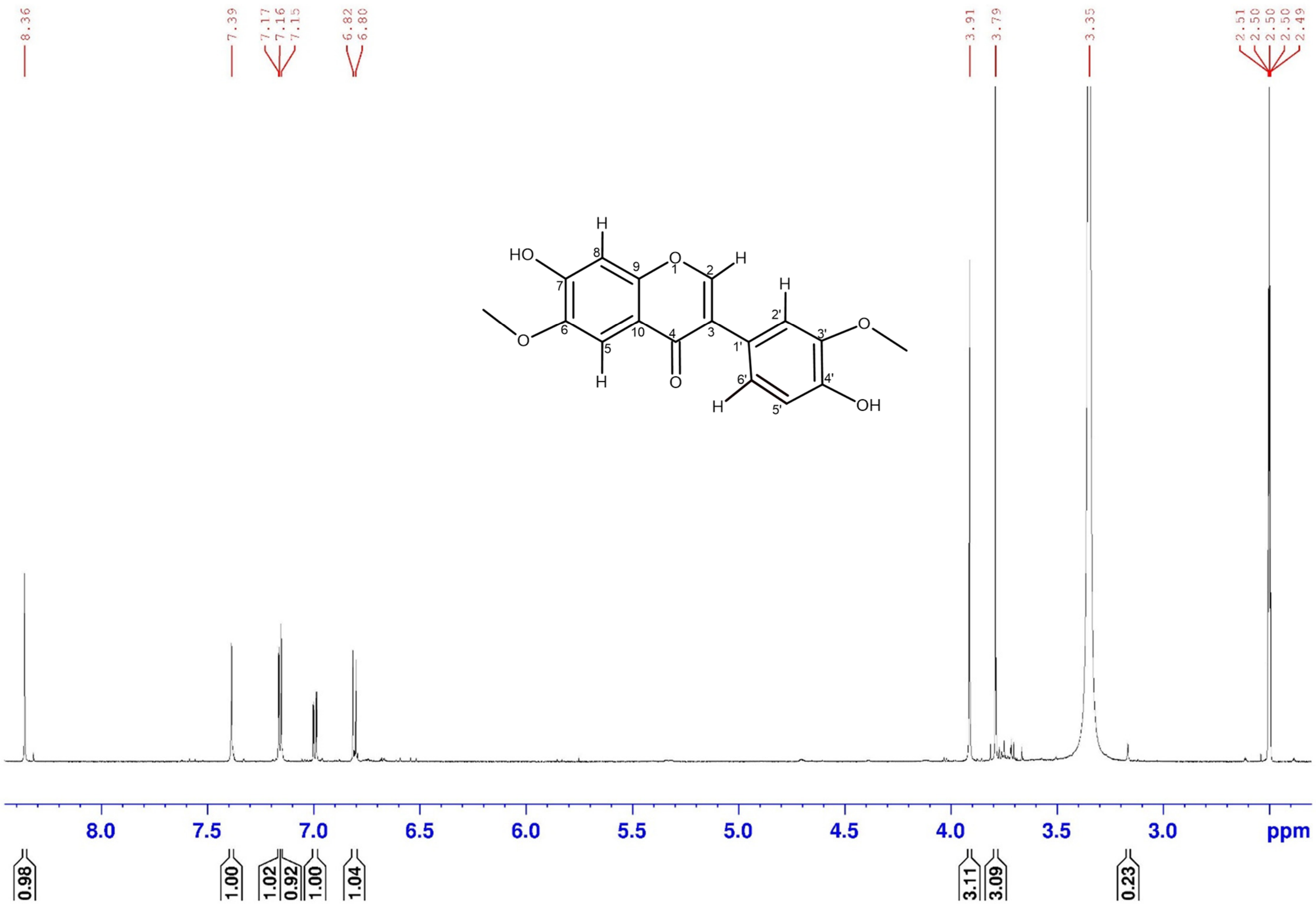

Figure S14. ^{1}H NMR spectrum of odoratin isomer (**2**).

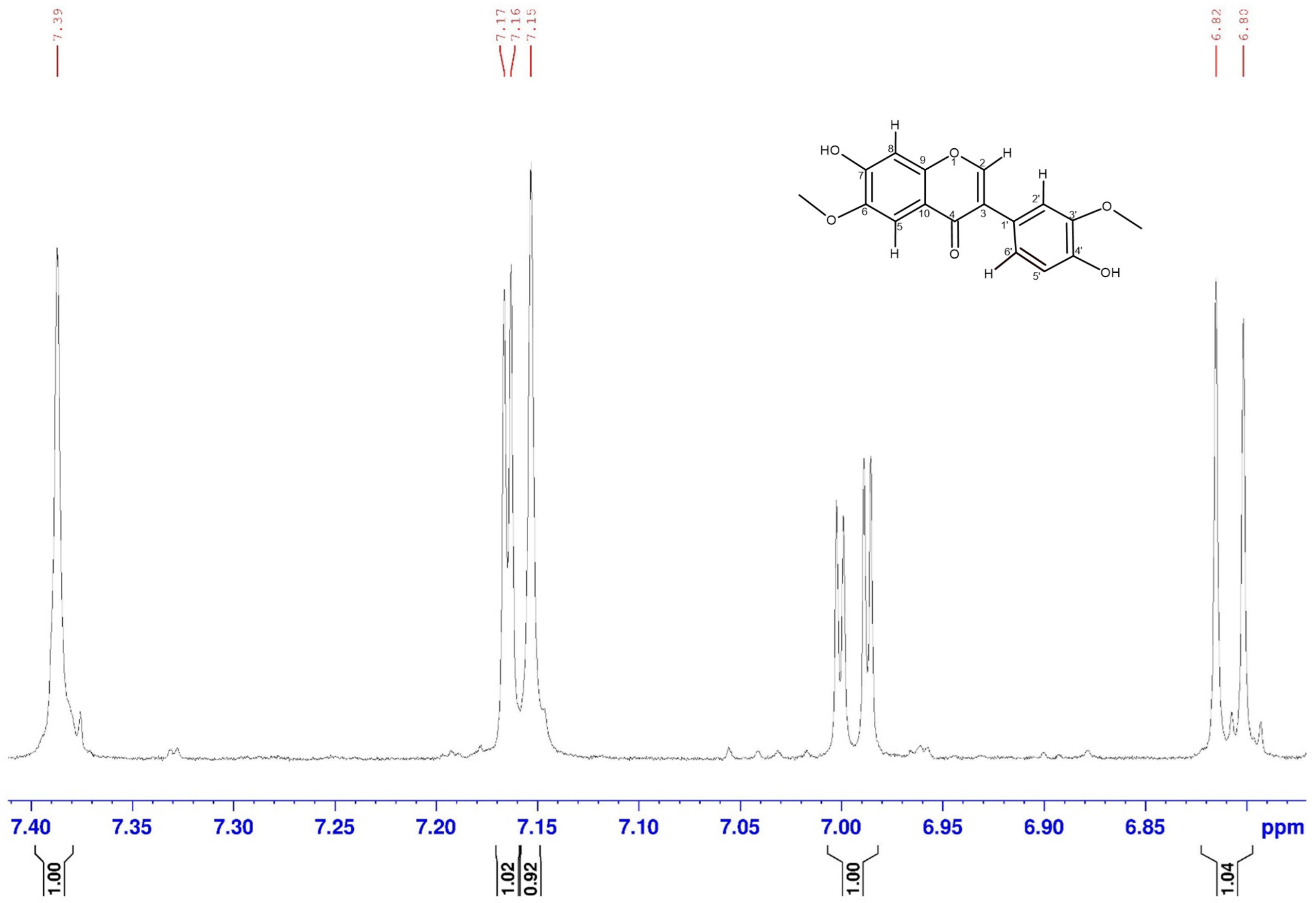

Figure S14a. ^{1}H NMR spectrum of odoratin isomer (**2**).

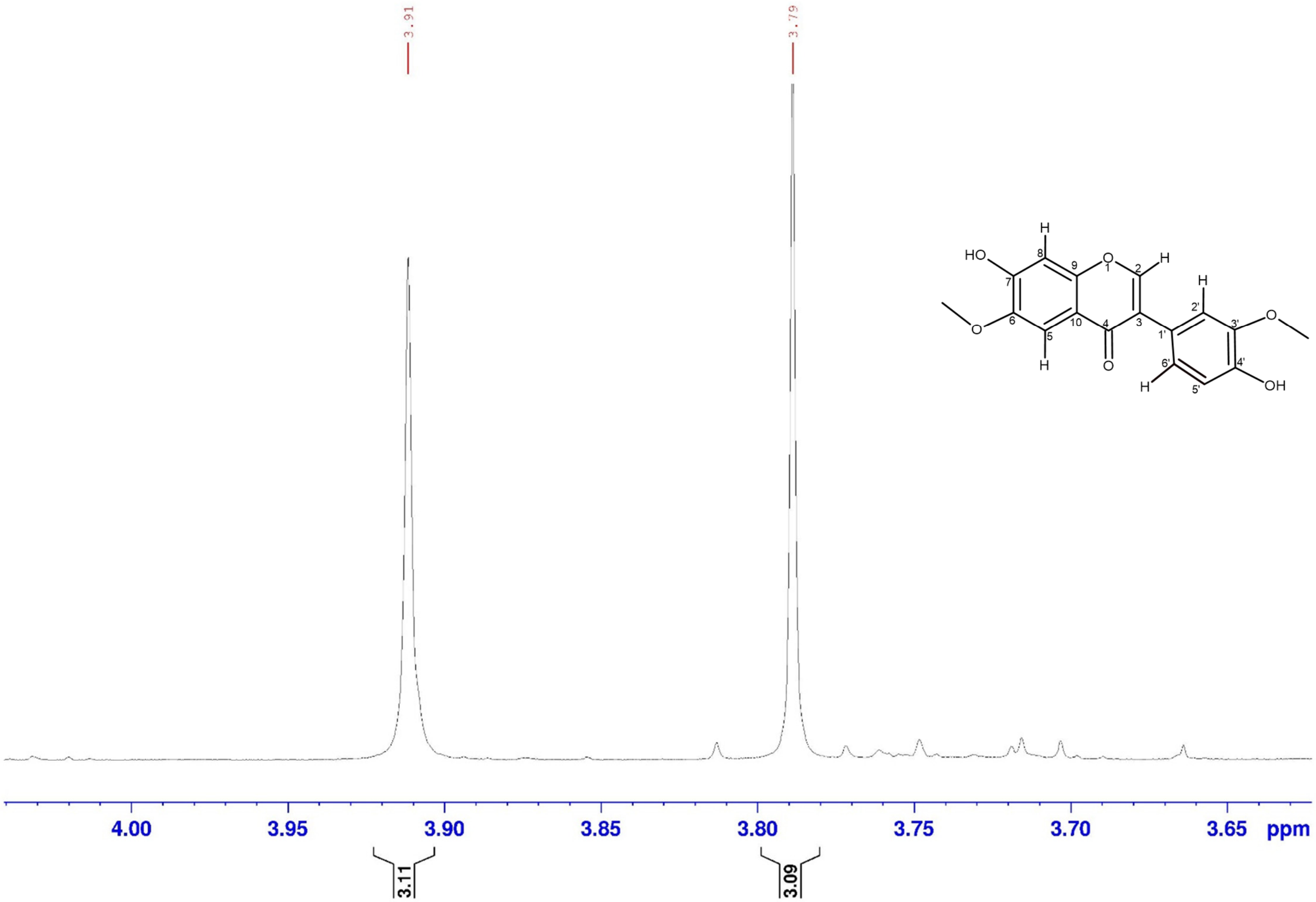

Figure S14b. ^{1}H NMR spectrum of odoratin isomer (**2**).

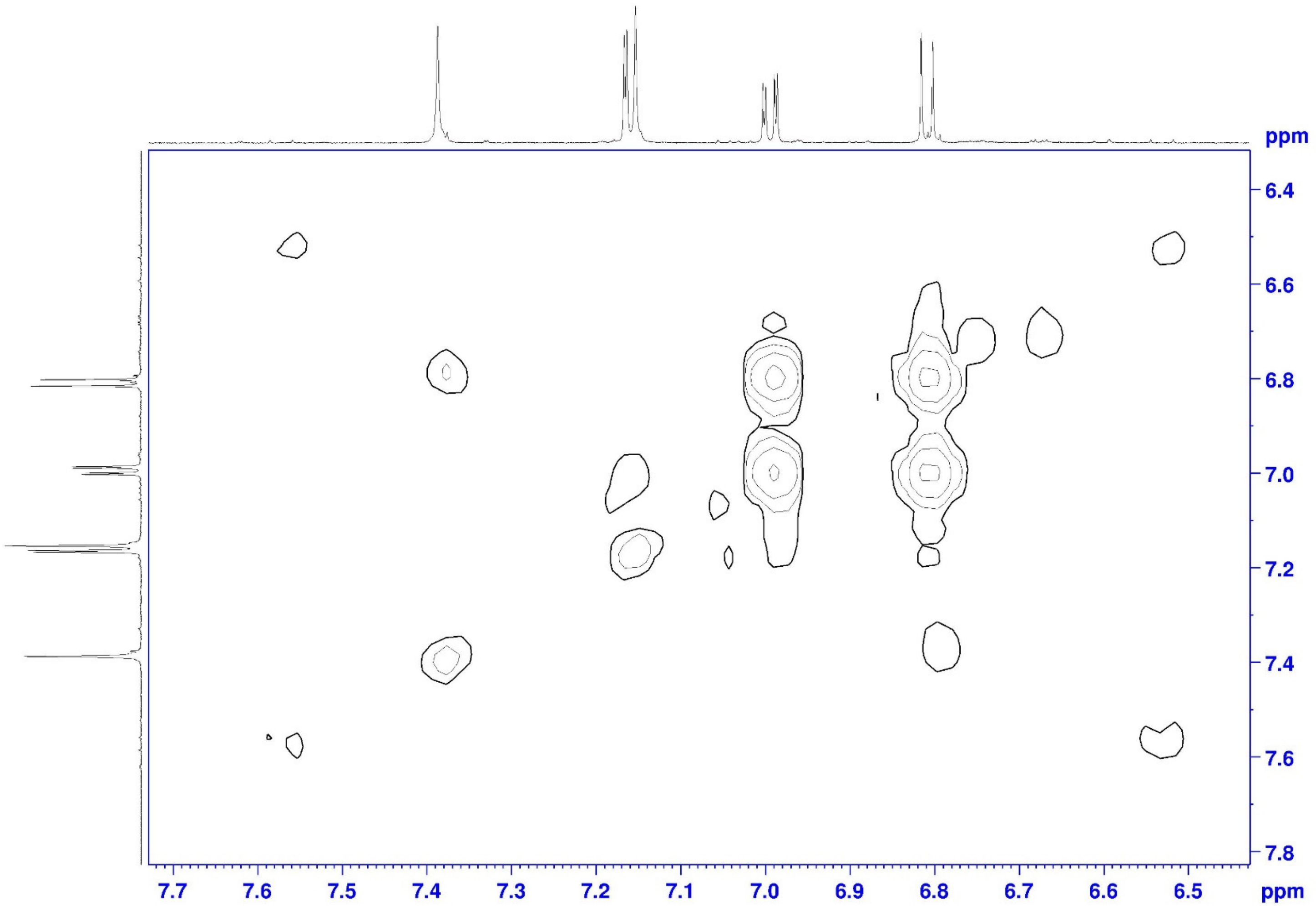

Figure S15. COSY spectrum of odoratin isomer (**2**).

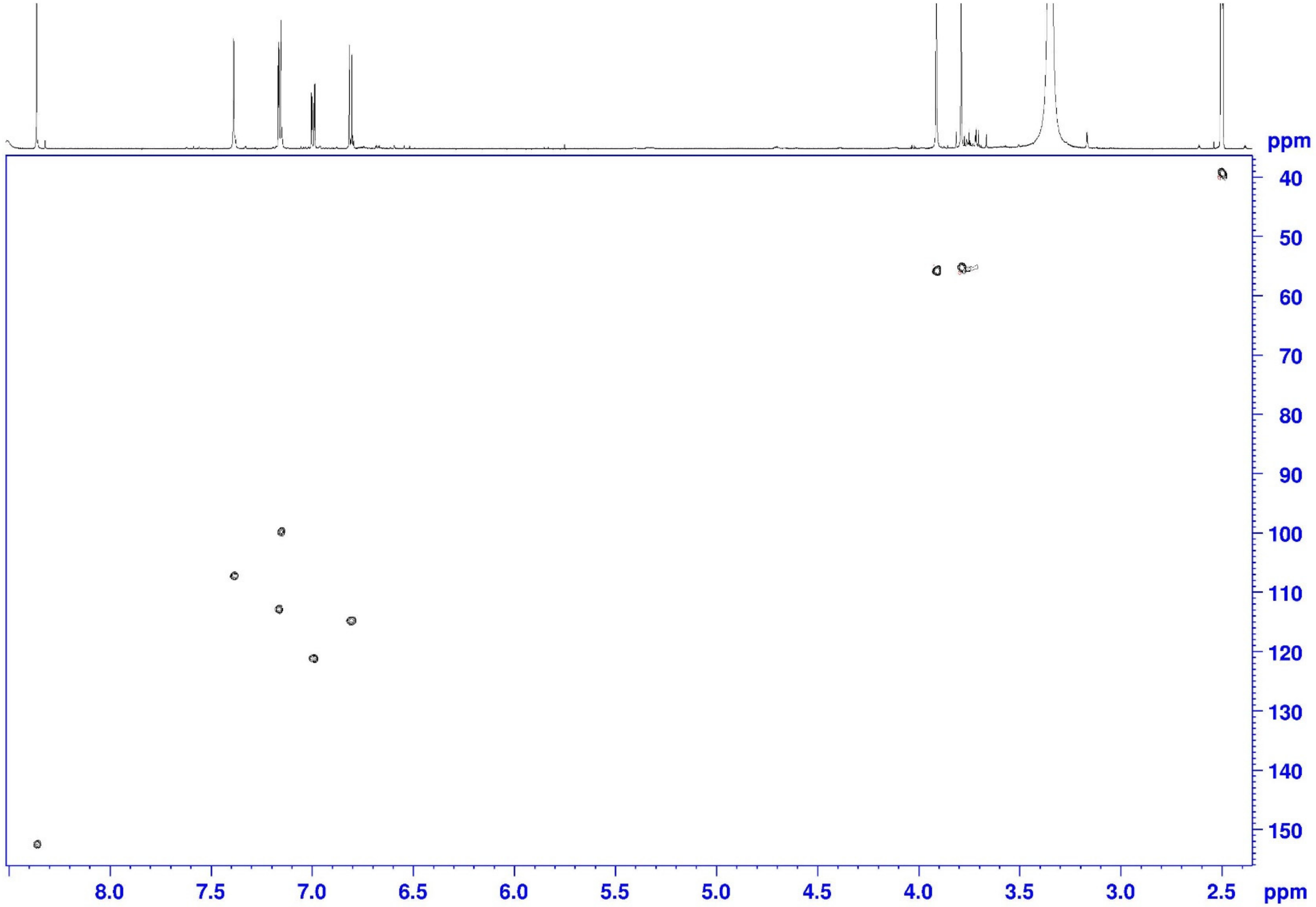

Figure S16. HSQC spectrum of odoratin isomer (**2**).

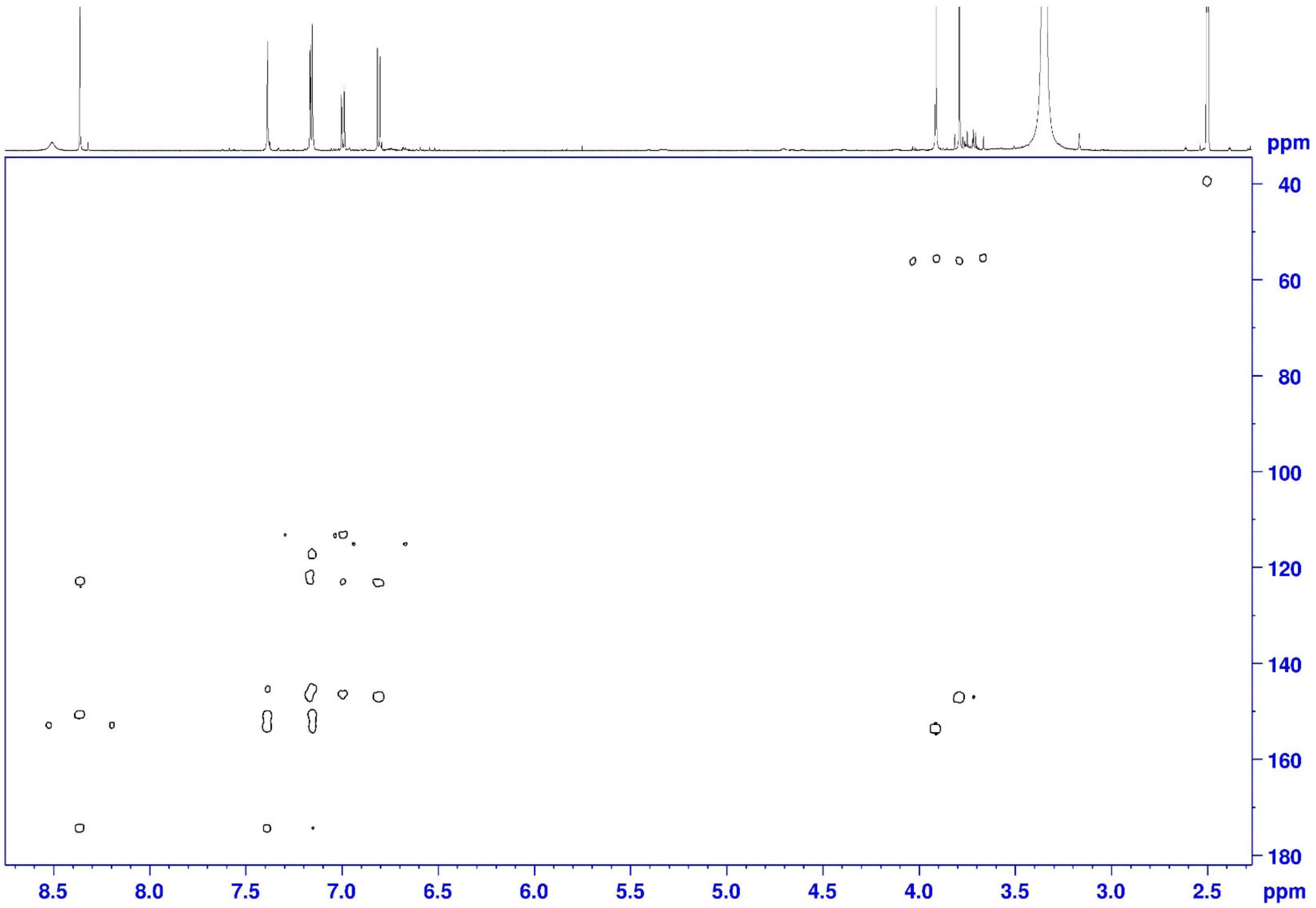

Figure S17. HMBC spectrum of odoratin isomer (**2**).

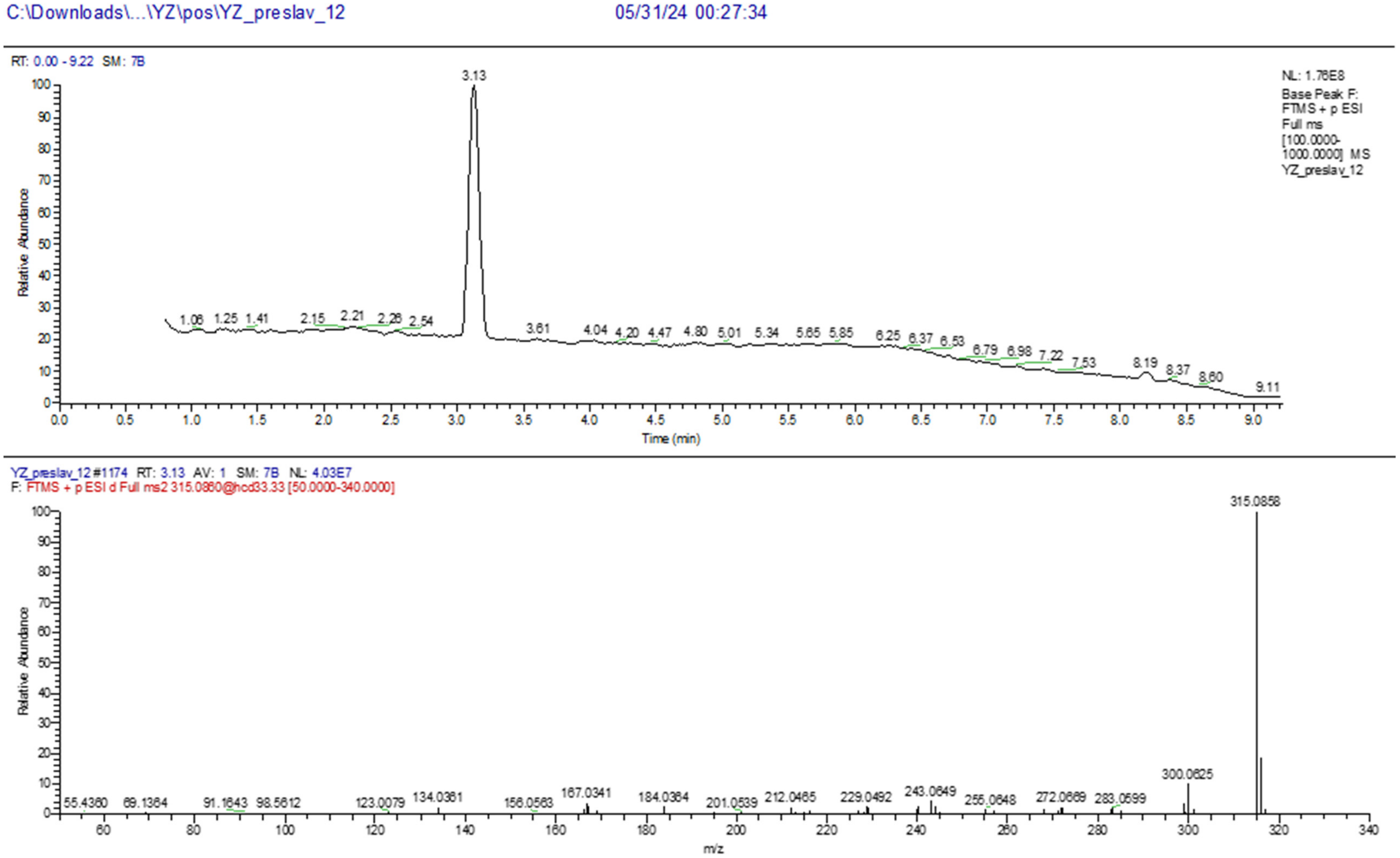

Figure S18. Chromatogram and MS spectrum of 6-hydroxy-3-(2-hydroxy-4-methoxyphenyl)-7-methoxy-4*H*-1-benzopyran-4-one (**6**)

Compound **6** was isolated as white amorphous powder (1.1 mg) and observed as protonated molecular ion with *m/z* 315.0858 corresponding to molecular formula$C_{17}H_{15}O_6^+$ (calcd. 315.0863) and retention time 3.13′.

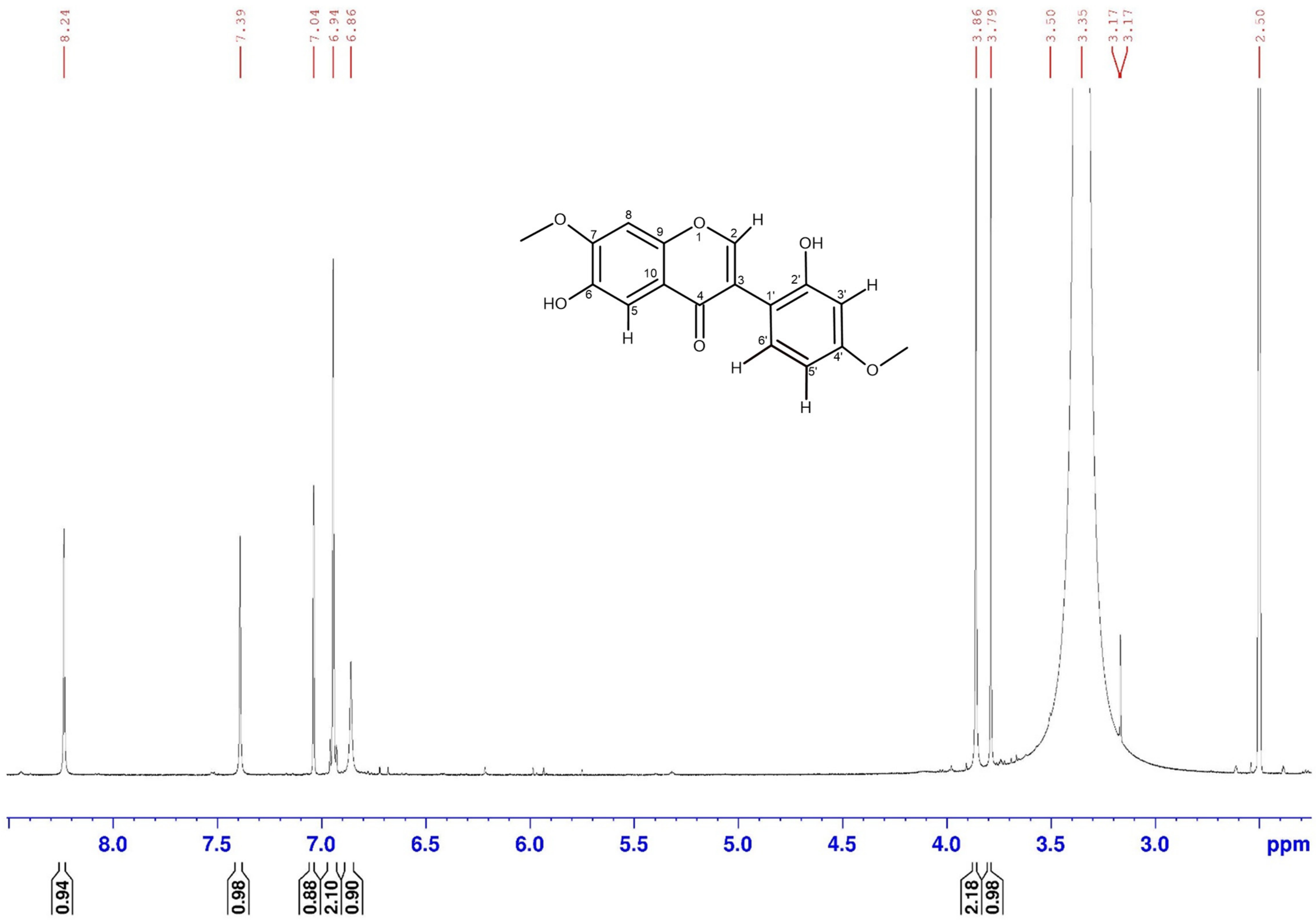

Figure S19. ^{1}H NMR spectrum of 6-hydroxy-3-(2-hydroxy-4-methoxyphenyl)-7-methoxy-4*H*-1-benzopyran-4-one (**6**)

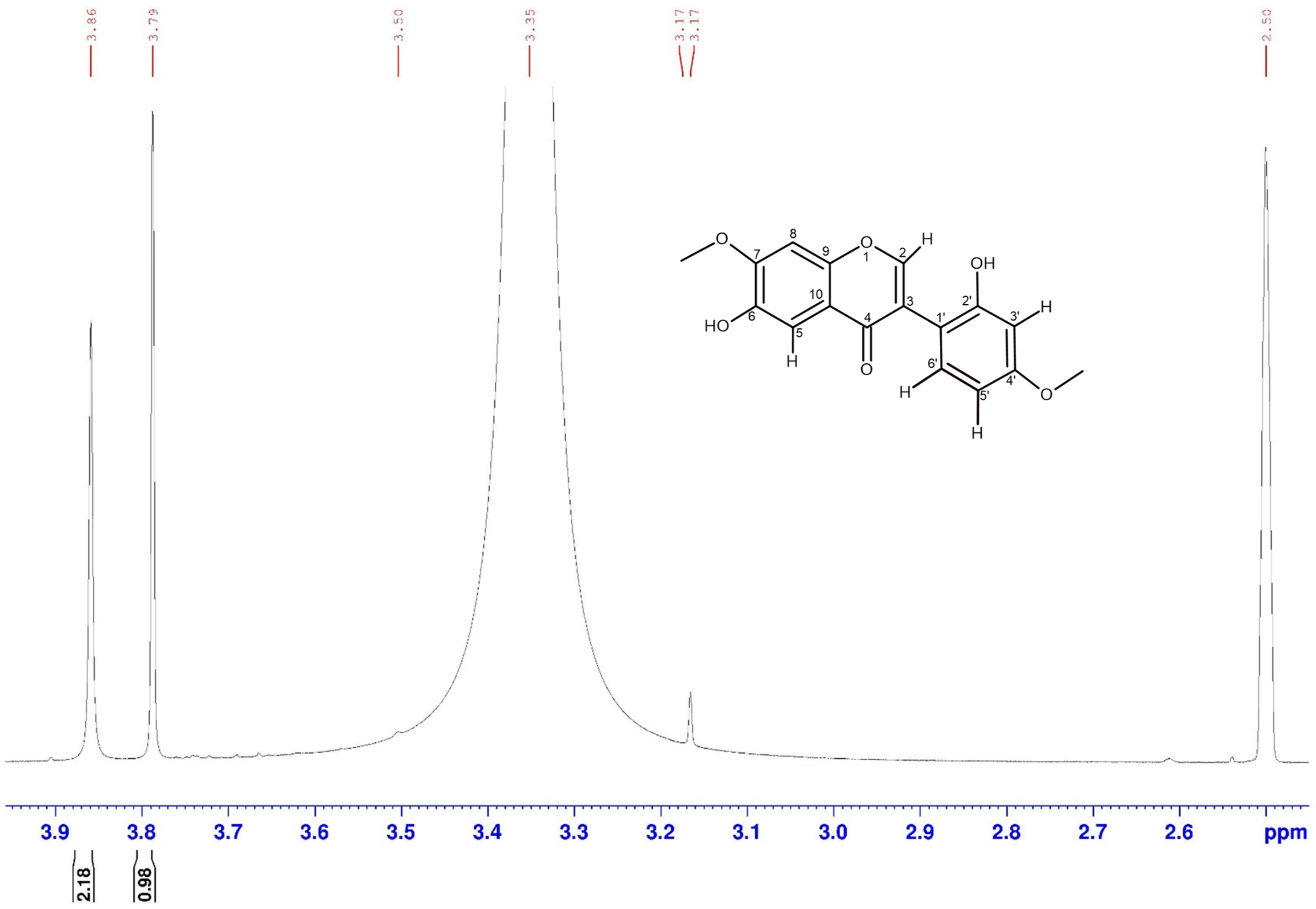

Figure S19a. ^{1}H NMR spectrum of 6-hydroxy-3-(2-hydroxy-4-methoxyphenyl)-7-methoxy-4*H*-1-benzopyran-4-one (**6**).

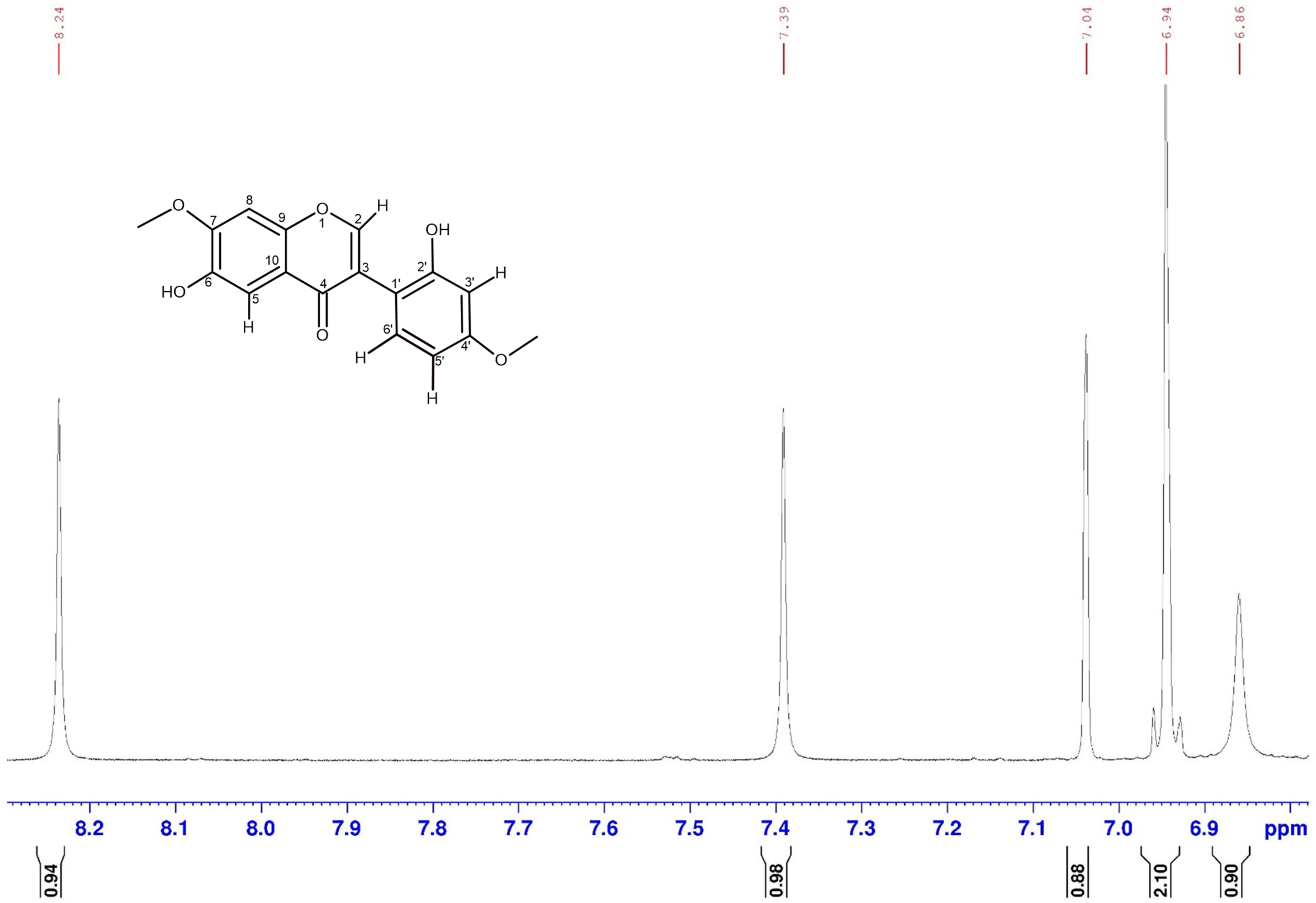

Figure S19b. ^{1}H NMR spectrum of 6-hydroxy-3-(2-hydroxy-4-methoxyphenyl)-7-methoxy-4*H*-1-benzopyran-4-one (**6**).

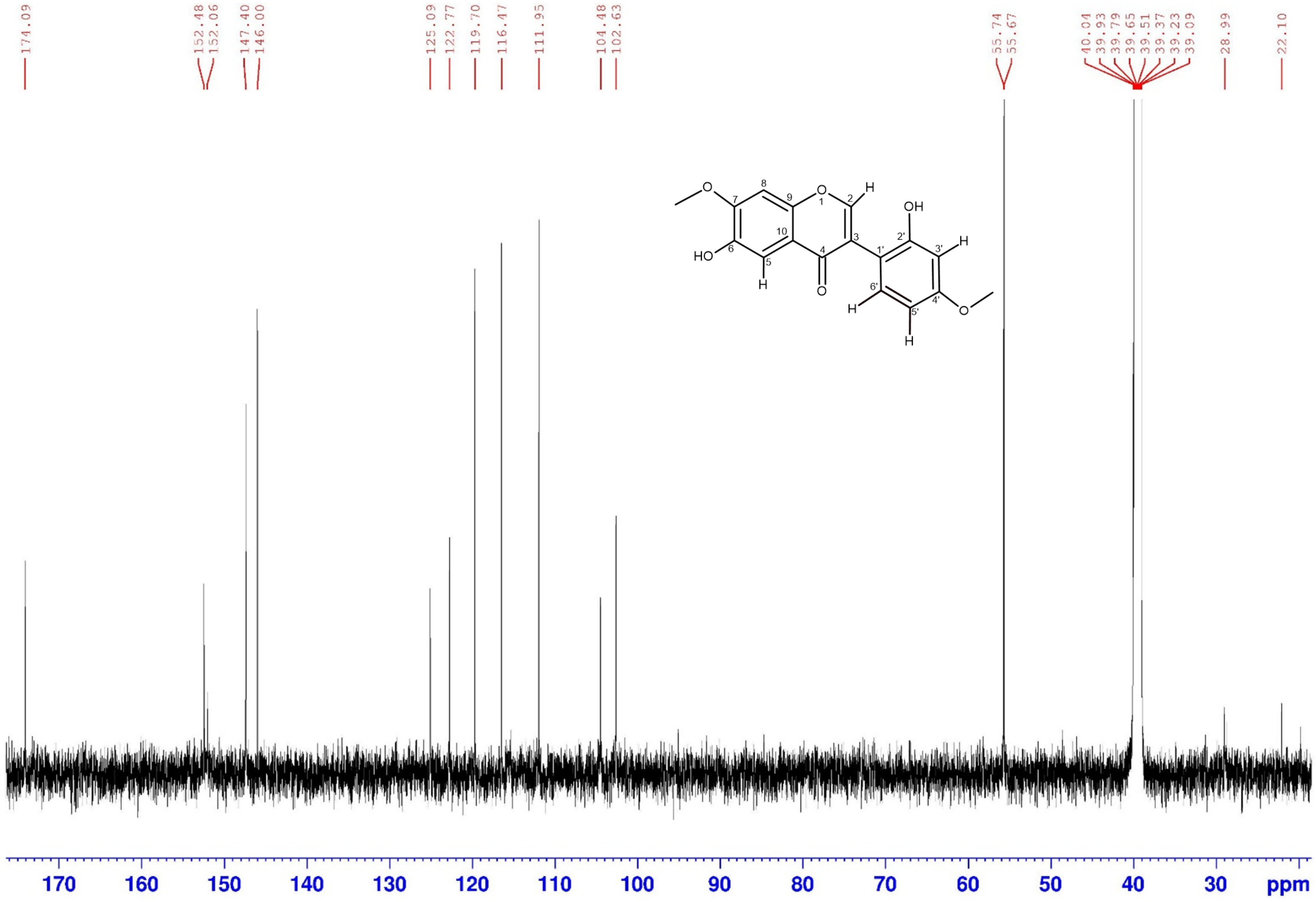

Figure S20. ^{13}C NMR spectrum of 6-hydroxy-3-(2-hydroxy-4-methoxyphenyl)-7-methoxy-4*H*-1-benzopyran-4-one (**6**).

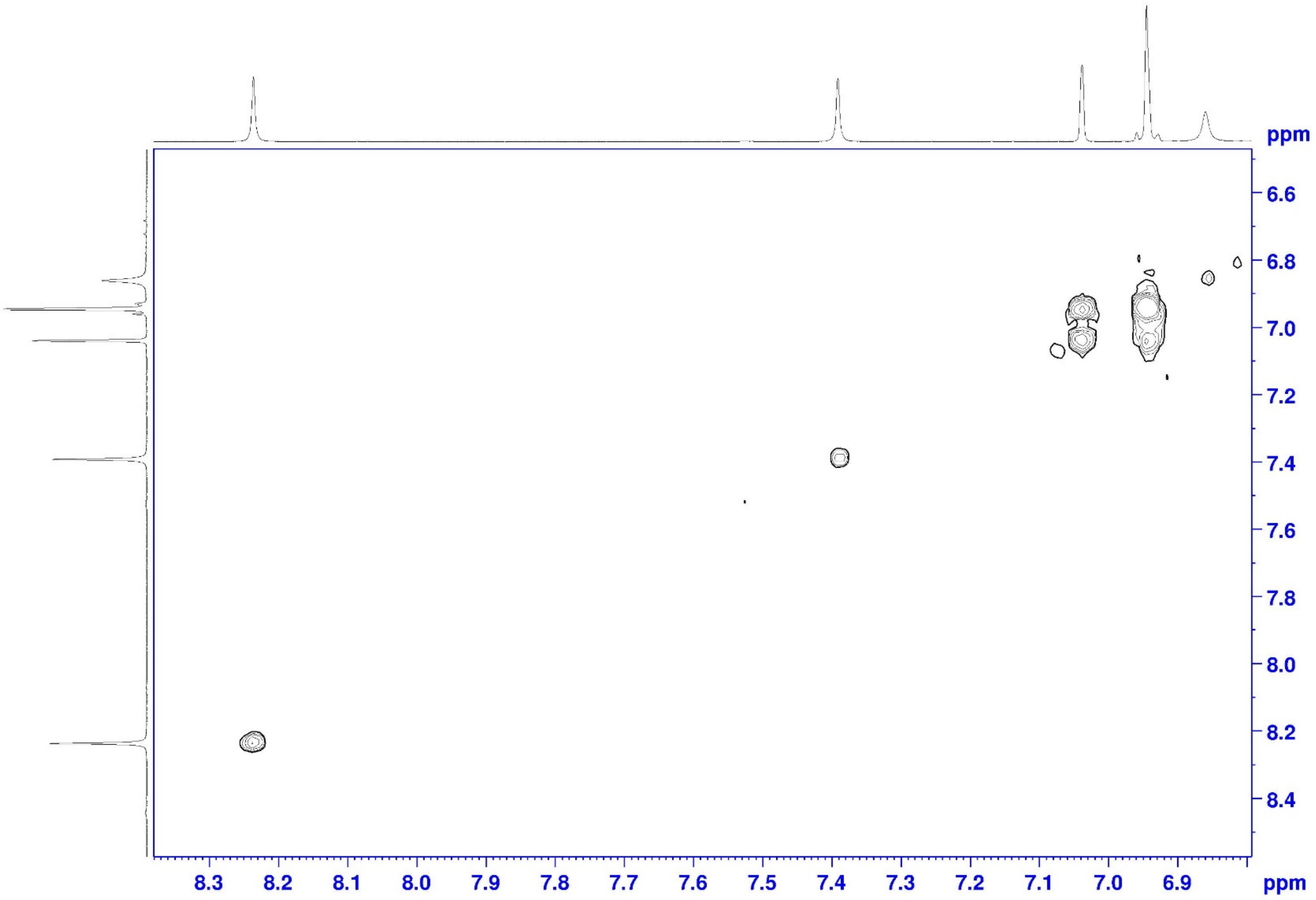

Figure S21. COSY spectrum of 6-hydroxy-3-(2-hydroxy-4-methoxyphenyl)-7-methoxy-4*H*-1-benzopyran-4-one (6).

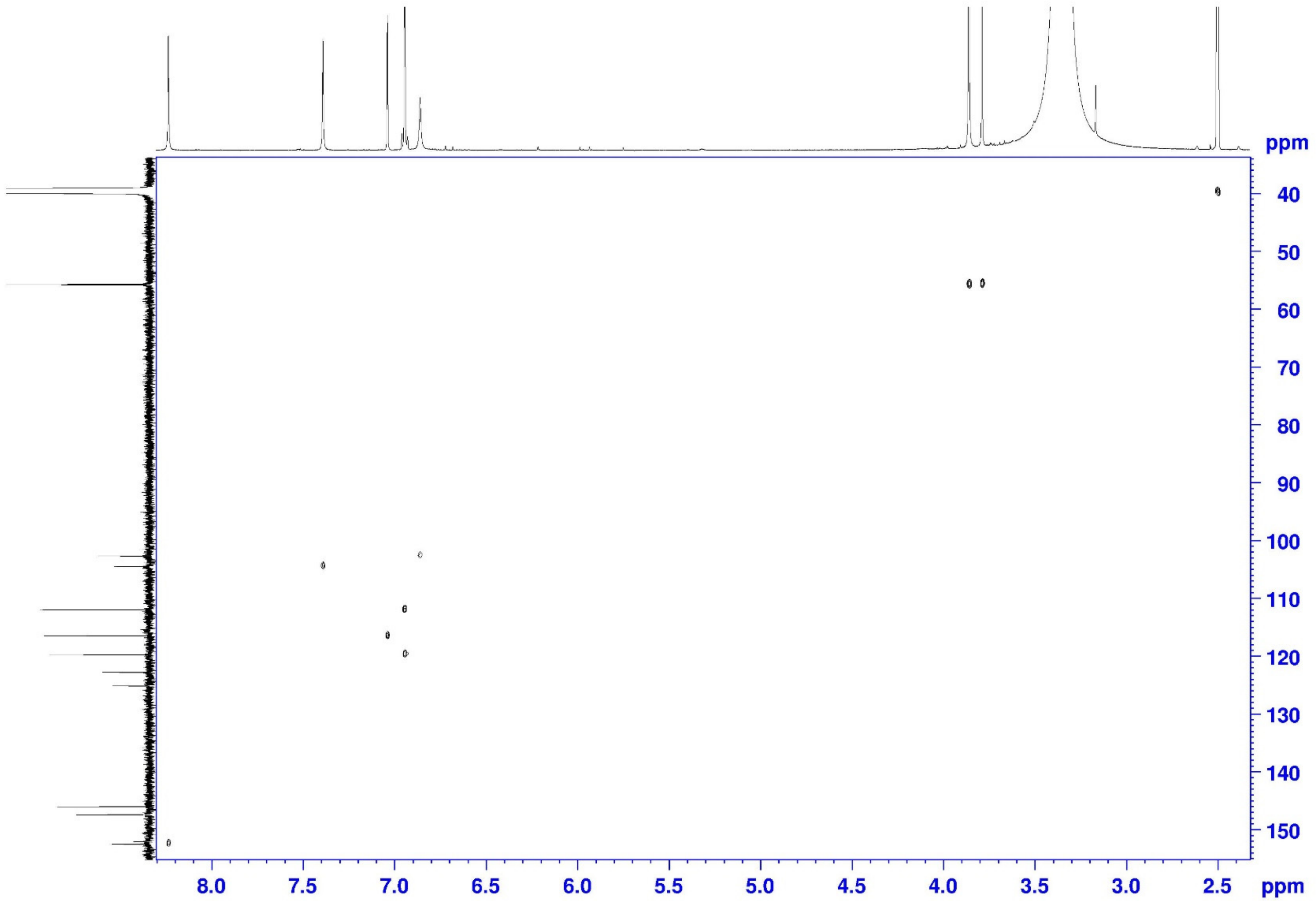

Figure S22. HSQC spectrum of 6-hydroxy-3-(2-hydroxy-4-methoxyphenyl)-7-methoxy-4*H*-1-benzopyran-4-one (6).

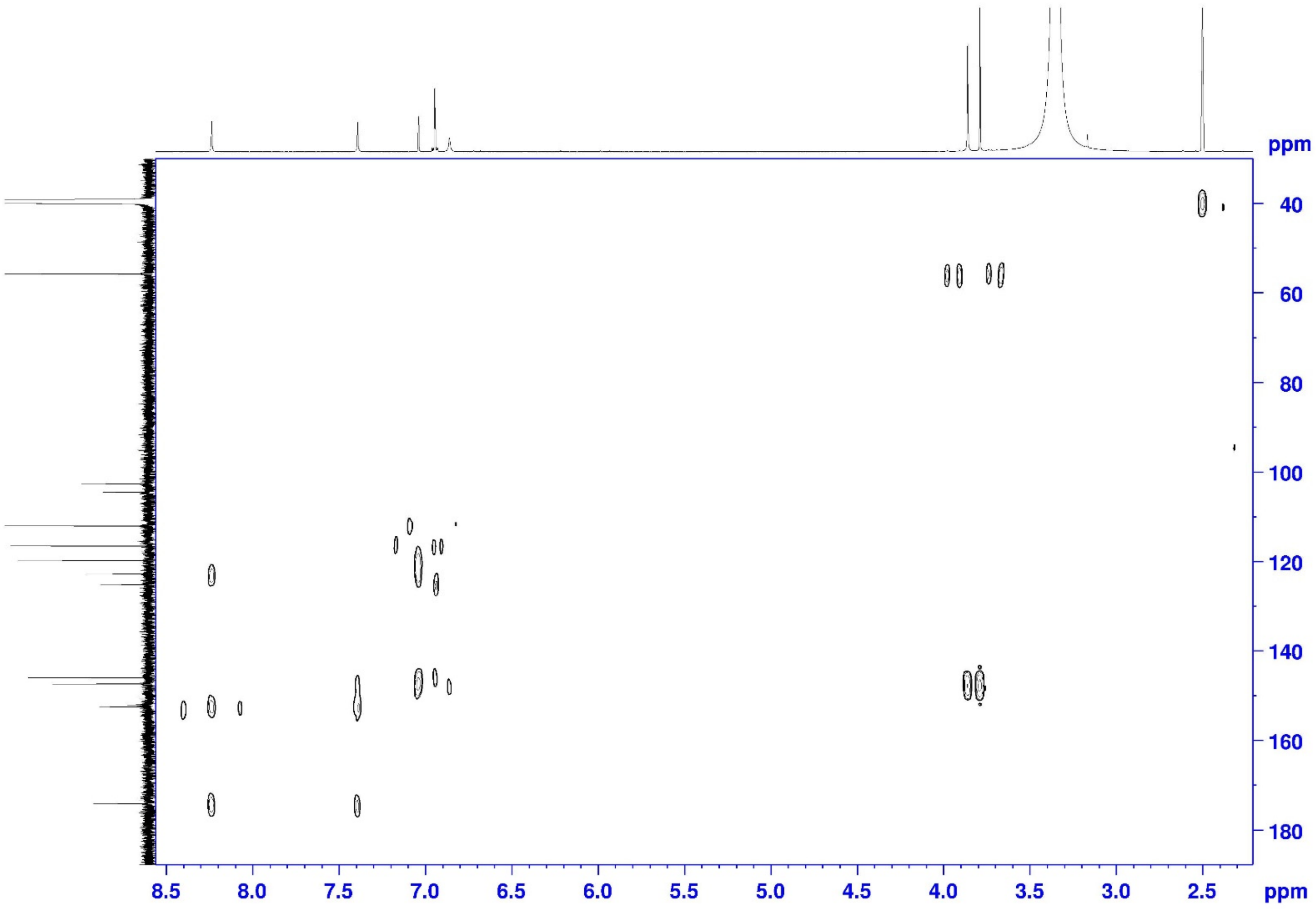

Figure S23. HMBC spectrum of 6-hydroxy-3-(2-hydroxy-4-methoxyphenyl)-7-methoxy-4*H*-1-benzopyran-4-one (6).

^{13}C and ^{1}H NMR data for compounds **1-14**.

Methyl ferulate
^{1}H NMR, (DMSO, 600 MHz) δ: 7.53 (1H, d, J=15.86, C-7), 7.27 (1H, s, C-2), 7.09 (1H, dd, J=8.21, 1.43, C-6), 6.75 (1H, d, J=8.10, C-5), 6.42 (1H, d, J=15.91, C-8), 3.68 (3H, s, OCH_3-, C-9).
^{13}C NMR, (DMSO, 600 MHz) δ: 167.15 (C, C-9), 148.28 (C, C-3), 147.98 (C, C-4), 144.92 (CH, C-7), 122.98 (C, C-6), 115.32 (CH, C-5), 113.28 (CH, C-8), 111.02 (CH, C-2), 50.87 (CH_3, -OCH_3, C-9).

Fujikinetin
^{1}H NMR, (DMSO, 600 MHz) δ: 8.46 (1H, s, HO, C-7), 8.22 (1H, s, C-2), 7.30 (1H, s, C-5), 7.14 (1H, d, J=1.77 Hz, C-6′), 7.03 (1H, dd, J=8.03, 1.69 Hz, C-2′), 6.95 (1H, d, J=8.03, C-3′), 6.72 (1H, s, C-8), 6.03 (2H, s, -OCH_2O-), 3.81 (3H, s, -OCH_3, C-6).
^{13}C NMR, (DMSO, 600 MHz) δ: 173.42 (C, C-4), 165.48 (C, C-7), 153.00 (C, C-9), 151.91 (CH, C-2), 148.42 (C, C-6), 147.03 (C, C-5′), 146.75 (C, C-4′), 126.54 (C, C-1′) 122.17 (C, C-3), 121.98 (C, C-2′), 109.29 (CH, C-6′), 107.75 (CH, C-3′), 103.70 (CH, C-5), 102.10 (CH, C-8), 100.59 (C, -OCH_2O-), 55.39 (CH_3, -OCH_3, C-6).

Pseudobaptigenin
^{1}H NMR, (DMSO, 600 MHz) δ: 8.28 (1H, s, C-2), δ 7.90 (1H, d J=8.73 Hz, C-5), δ 7.13 (1H d, J=1.80, C-6′), 7.04 (1H, dd, J=8.18, 1.72), 6.95 (1H, d, J=8.02 Hz, C-3′), 6.87 (1H, d, J=8.78, C-6), 6.78 (1H, 6.78, s, C-8), 6.03(2H, s, -OCH_2O-).
^{13}C NMR, (DMSO, 600 MHz) δ: 174.36 (C, C-4), 164.86 (C, C-7), 153.12 (CH, C-2), 152.69 (C, C-9), 146.96 (C, C-5′), 146.81 (C, C-4′), 126.96 (CH, C-5), 126.05 (C, C-1′), 122.98 (C, C-3), 122.36 (CH, C-2′), 116.21 (CH, C-6), 115.60 (C, C-10), 108.07 (CH, C-3′), 102.04 (CH, C-8), 100.99 (C, -OCH_2O-).

Formononetin
^{1}H NMR (DMSO, 600 MHz) δ: 8.48 (1H, s, HO, C-7), 8.27 (1H, s, C-2), 7.89 (1H, d, J=8.80 Hz), 7.49 (2H, dt, C-3′,5′)′, C-2′, C-6′), 6.97 (2H, dt, C-2′, C-6′), 6.86 (1H, d, J= 8.80, C-6), 6.77 (1H, s, C-8), 3.78 (3H, s, -OCH3, C4′).
^{13}C NMR, (DMSO, 600 MHz) δ: 174.60 (C, C-3), 165.70 (C, C-7), 159.00 (C, C-4′), 157.90 (C, C-9), 152.77 (CH, C-1), 130.08 (CH, C-2′), 130.08 (CH, C-6′), 126.93 (C, C-5), 124.60 (C, C-1′), 123.10 (C, C-1′), 116.00 (CH, C-6), 115.50 (C, C-10), 113.60 (C, C-3′), 113.60 (C, C-5′), 102.08 (C, C-8), 55.00 (C, OCH3, C-4′).

3′-methoxydaidzein
^{1}H NMR (DMSO, 600 MHz) δ: 8.25 (1H, s, C-2), 7.89 (1H, d, J=8.69, C-6), 7.15 (1H, d, J=2.02 Hz, 6.97 (1H, dd, J=8.13, 2.03 Hz), 6.83 (1H, d, J=9.00, C-5), 6.81 (1H, d, J= 8.24 Hz, C-5′), 6.72 (1H, s, C-8), 3.78 (3H, s, -OCH_3, C-3′).
^{13}C NMR (DMSO, 600 MHz) δ: 174.30 (C, C-5), 165.19 (C, C-6), 157.90 (C, C-9), 152.48 (CH, C-2), 147.28 (C, C-3′), 146.40 (C, C-4′), 126.81 (CH, C-7), 123.45 (C, C-1′), 121.90 (C, C-1′),

121.30 (CH, C-6′), 115.67 (CH, C-8), 114.99 (CH, C-5′), 113.14 (CH, C-2′), 101.73 (CH, C-5), 55.42 (-OCH_3, C-3′) n.o. (C, C-10).

6,4′-dimethoxy-7,2′-dihydroxy isoflavone

^{1}H NMR, (DMSO, 600 MHz) :δ 8.06 (1H, s, C-2), 7.19 (1H, s, C-5), 7.13 (1H, dt, C-6′), 6.44 (1H, d, J=1.96, C-3′), 6.43 (1H, dd, J=7.20, 2.68, C-5′), 6.43 (1H, s, C-8), 3.76 (3H, s, OCH_3, C-6), 3.72 (3H, s, OCH_3, C-4′).

^{13}C NMR, (DMSO, 600 MHz) δ: 174.40 (C, C-4), 165.33 (C, C-7), 160.80 (C, C-4′), 157.00 (C, C-2′), 154.50 (C, C-9), 152.52 (CH, C-2), 150.90 (C, C-6), 131.00 (CH, C-6′), 121.20 (C, C-3), 113.50 (C, C-1′), 104.84 (CH, C-5′), 102.42 (CH, C-3′), 102.10 (CH, C-5), 100.91 (CH, C-8), 55.03 (C, -OCH_3, C-6), 54.81 (C, -OCH_3, C-4′).

Onogenin

^{1}H NMR (DMSO, 600 MHz) δ: 7.66 (1H, d, J=8.72, C-5), 6.82 (1H, s, C-5′), 6.71 (1H, s, C-2′), 6.51 (1H, d, J=8.68, 2.20 Hz, C-6), 6.33 (1H, d, J=2.02 Hz, C-8), 5.95 (2H, dd, J= 5.40, 0.69 Hz, -OCH_2O-), 4.51 (1H, t, C-3b), 4.40 (1H, dd, J=11.05, 5.43 Hz, C-3a), 4.18 (1H, dd, J= 12,94, 5.52 Hz, C-2), 3.66 (3H, s,-OCH_3, C-6′).

^{13}C NMR (DMSO, 600 MHz) δ 190.23 (C, C-4), 163.24 (C, C-10), 152.53 (C, C-6′), 147.14 (C, C-3′), 128.98 (CH, C-5), 115.78 (C, C-4′), 113.84 (C, C-7), n.o. (C, C-9), 110.70 (CH, C-6), 109.78 (CH, C-2′), 102.40 (CH, C-8), 101.08 (C, -OCH_2O-), 95.75 (CH, C-5′), 70.23 (CH2, C-3), 46.94 (CH, C-2).

Sayanedine

^{1}H NMR (DMSO, 600 MHz) δ**:** 8.41 (1H, s, C-2), 8.03 (1H, d, J=8.87 Hz, C-5), 7.17 (1H, d, J=2.00), 7.16 (1H, d, J=2.42, C-8), 7.08 (1H, dd, J=8.89, 2.35, C-6), 7.01 (1H, dd, J=8.16, 2.00 Hz, C-6′), 6.82 (1H, d, J=8.02 Hz, C-5′), 3.91 (3H, s, OCH_3, C-7), 3.79 (3H, OCH_3, C-3′).

^{13}C NMR (DMSO, 600 MHz) δ: 174.82 (C, C-4), 163.78 (C, C-7), 157.42 (C, C-9), 153.08 (CH, C-2), 147.24 (C, C-3′), 146.50 (C, C-4′), 126.74 (CH, C-5), 123.63 (C, C-1′), 121.78 (C, C-3), 121.43 (CH, C-6′), 114.93 (CH, C-5′), 114.67 (CH, C-6), 113.00 (CH, C-2′), 100.40 (CH, C-8), 55.82 (-OCH_3, C-7), 55.40 (-OCH_3, C-3′).

Cajanin

^{1}H NMR (DMSO, 600 MHz) δ:12.99 (1H, s, C-5), 8.22 (1H, s, C-2), 6.98 (1H, d, J=8.24 Hz), 6.64 (1H, d, J=2.27 Hz, C-8), 6.40 (1H, d, J=2.31 Hz, C-6), 6.36 (1H, d, J=2.41 Hz, C-3′), 6.26 (1H, dd, J=8.27, 2.41, C-5′), 3.86 (3H, -OCH_3, C-7).

^{13}C NMR (DMSO, 600 MHz) δ: 180.65 (C, C-4), 165.13 (C, C-7), 161.79 (C, C-5), 158.66 (C, C-4′), 157.55 (C, C-9), 156.43 (C, C-2′), 155.40 (CH, C-2), 132.20 (CH, C-6′), 120.74 (C, C-3), 108.44 (C, C-1′), 106.25 (CH, C-5′), 105.49 (C, C-10), 102.64 (C, C-3′), 97.92 (C, C-6), 92.39 (C, C-8), 56.10 (-OCH3, C-7).

Afrormosin

^{1}H NMR (DMSO, 600 MHz) δ: 8.44 (1H, s, C-7), 8.26 (1H, s, C-2), 7.50 (2H, dt, C-2′, C-6′), 7.36 (1H, s, C-5), 6.98 (2H, dt, C-3′), 6.98 (2H, dt, C-3′, C-5′), 6.83 (1H, s, C-8), 3.84 (3H, s, -OCH_3, C-6), 3.78 (3H, s, -OCH_3, C-4′).

^{13}C NMR (DMSO, 600 MHz) δ: 174.40 (C, C-4), 158.80 (C, C-4′), 152.49 (C, C-7), 152.35 (CH, C-2),152.30 (C, C-9), 147.80 (C, C-6), 130.06 (CH, C-2′), 130.06 (CH, C-6′), 125.00 (C, C-3), 122.50 (C, C-1′), 113.57 (CH, C-3′), 113.57 (CH, C-5′), 104.23 (CH, C-5), 102.60 (CH, C-8), 55.66 (C, -OCH_3, C-6).

Trifoliol

^{1}H NMR (DMSO, 600 MHz) δ: 7.74 (1H, d, J=8.41 Hz, C-5), 7.13 (1H, d, J=1.99 Hz, C-8), 6.94 (1H, dd, J=8.47, 1.99 Hz, C-6), 6.86 (1H, s, C-4′), 6.51 (1H, s, C-2′), 3.88 (3H, s, -OCH_3-, C-3′)

^{13}C NMR (DMSO, 600 MHz) δ: 178.21 (C, C-2), 164.68 (C, C-3′), 164.55 (C, C-5′), 156.54 (C, C-7), 154.60 (C, C-3), 150.05 (C, C-4b), 121.22 (C, C-5), 113.98 (C, C-6), 113.14 (C, C-4a), 103.96 (C, C-6′), 98.93 (C, C-2′), 98.90 (C, C-8), 97.54 (C, C-4), 93.92 (C, C-4′), 56.28 (C, -OCH_3-, C-3′)

Maackiain

^{1}H NMR (DMSO, 600 MHz) δ: 7.23 (1H, d, J=8.55 Hz, C-1), 6.96 (1H, s, C-10), 6.51 (d, J=2.42 Hz, C-7), 6.45 (1H, dd, J=2.46, 8.38 Hz, C-2) ,6.25 (1H, d ,J=2.42 Hz ,C-4), 5.70 (1H, d, J=0.91, C-8), 5.66 (1H, d, J=0.91 Hz, C-9), 5.50 (1H, d, J=7.10, C-11a), 4.22 (1H, m, C-6), 3.59 (1H, m, C-6), 3.55 (1H, m, C-6a)

^{13}C NMR (DMSO, 600 MHz) δ: 158.74 (C, C-3), 156.33 (C, C-4a), 153.74 (C, C-10a), 147.51 (C, C-9), 141.07 (C, C-8), 132.03 (CH, C-1), 118.46 (C, C-6b), 109.70 (C, C-2), 105.40 (C, C-10), 102.84 (CH, C-4), 93.23 (CH, C-7), 77.99 (C, C-11a), 65.78 (C, C-6)

Isomer of odoratin

UV (MeOH) (log ε) 208 (4.67), 263 (4.49), 325 (4.15)

^{1}H NMR (DMSO, 600 MHz) δ: 8.36 (1H**,** s, C-4), 7.38 (1H, s, C-5), 7.16 (1H, d, J=2.00 Hz, C-2′), 7.15 (1H, s, C-8), 6.99 (1H, dd, J= 8.18, 2.05 Hz, C-6′), 6.80 (1H, d, J=8.20 Hz, C-5′), 3.91 (3H, -OCH_3-, C-6), 3.78 (3H, -OCH_3-, C-3′)

^{13}C NMR (DMSO, 600 MHz) δ: 174.37 (C, C-4), 153.80 (C, C-6), 152.65 (CH, C-2), 150.80 (C, C-9), 147.40 (C, C-3′), 146.50 (C, C-4′), 145.70 (C, C-7), 123.08 (C, C-1′), 121.29 (C, C-6′), 117.50 (C, C-10), 114.92 (CH, C-5′), 113.04 (CH, C-2′), 107.33 (CH, C-5), 100.09 (CH, C-8), 55.95 (CH_3, -OCH_3-, C-6), 55.48 (CH_3, -OCH_3, C-3′)

6-hydroxy-3-(2-hydroxy-4-methoxyphenyl)-7-methoxy-4*H*-1-benzopyran-4-one

UV (MeOH) (log ε) 202 (6.69), 220 (6.49), 259 (6.30), 285 (6.09), 319 (6.00)

^{1}H NMR (DMSO, 600 MHz) δ: 8.23 (1H, s, C-2), 7.39 (1H, s, C-5), 7.03 (1H, s, C-5′), 6.94 (1H, s, C-3′), 6.94 (1H, s, C-6′), 6.86 (1H, s, C-8)

^{13}C NMR (DMSO, 600 MHz) δ: 174.10 (C, C-4), 152.60 (C, C-9), 152.47 (C, C-2), 148.48 (C, C-6′), 147.53 (C, C-7), 147.50 (C, C-4′), 146.10 (C, C-2′), 125.10 (C, C-3), 122.90 (C, C-1′), 119.80 (C, C-3′), 116.59 (CH, C-5′), 112.01 (CH, C-6′), 104.52 (CH, C-5)
